# The Total Solubility of the Co-Solubilized PAHs with Similar Structures Indicated by NMR Chemical Shift

**DOI:** 10.3390/molecules26092793

**Published:** 2021-05-10

**Authors:** Tao Chen, Xin Hu, Zhong Chen, Xiaohong Cui

**Affiliations:** Department of Electronic Science, Fujian Provincial Key Laboratory of Plasma and Magnetic Resonance, State Key Laboratory of Physical Chemistry of Solid Surfaces, Xiamen University, Xiamen 361005, China; 33320181150283@stu.xmu.edu.cn (T.C.); 33320191150287@stu.xmu.edu.cn (X.H.); chenz@xmu.edu.cn (Z.C.)

**Keywords:** polycyclic aromatic hydrocarbon, co-solubilization, surfactant, NMR, total solubility

## Abstract

The synergism/inhibition level, solubilization sites and the total solubility (S_t_) of co-solubilization systems of phenanthrene, anthracene and pyrene in Tween 80 and sodium dodecyl sulfate (SDS) are studied by ^1^H-NMR, 2D nuclear overhauser effect spectroscopy (NOESY) and rotating frame overhauser effect spectroscopy (ROESY). In Tween 80, inhibition for phenanthrene, anthracene and pyrene is observed in most binary and ternary systems. However, in SDS, synergism is predominant. After analysis, we find that the different synergism or inhibition situation between Tween 80 and SDS is related to the different types of surfactants used and the resulting different co-solubilization mechanisms. In addition, we also find that three polycyclic aromatic hydrocarbons (PAHs) have similar solubilization sites in both Tween 80 and SDS, which are almost unchanged in co-solubilization systems. Due to the similar solubilization sites, the chemical shift changes of surfactant and PAH protons follow the same pattern in all solubilization systems, and the order of chemical shift changes is consistent with the order of changes in the St of PAHs. In this case, it is feasible to evaluate S_t_ of PAHs by chemical shift. In both Tween 80 and SDS solutions, the ternary solubilization system has relatively high S_t_ rankings. Therefore, in practical applications, a good overall solubilization effect can be expected.

## 1. Introduction

Polycyclic aromatic hydrocarbons (PAHs) compounds are a type of complex organic pollutants with fused ring structures containing at least two benzene rings. These compounds are mainly produced by synthetic means, such as the incomplete combustion of organic materials, the extensive use of fossil fuels and various industrial activities of human beings [1,2]. Due to their strong hydrophobicity, most PAHs are hard to decompose, and eventually deposited in the soil and exist in the environment extensively and persistently [3,4,5]. PAHs are carcinogenic, toxic and mutagenic, which can cause serious pollutions to humans and ecosystems [6,7,8]. Therefore, there is a great demand in the removal of PAHs from contaminated soil or sediment water, and various methods have been developed to remove PAHs, such as chemical, physical, microbial or phytoremediation, surfactant enhanced remediation (SER) methods [9,10,11,12,13,14,15,16,17,18,19,20,21]. SER methods are based on the amphiphilic properties of surfactants. Because they have both hydrophilic head groups and hydrophobic tails, surfactants can aggregate to form micelles above the critical micelle concentration (CMC), which can create a hydrophobic environment in aqueous solutions and make the hydrophobic compounds wrapped in micelles. Since the solubility of hydrophobic compounds in micellar solutions can be several hundred times higher than that in water, SER has been evaluated as a promising method for the remediation of PAHs [22].

In recent years, many studies have been carried out on the solubilization of PAHs by surfactants [23,24,25,26,27,28,29]. Aryal et al. reported that a single surfactant solution above CMC can enhance the solubility of phenanthrene (Phe) and pyrene (Pyr) in water, and the solubility was linearly proportional to the surfactant concentration [24]. Sales et al. found that the mixed micelles of Tween 80 and sodium laurate were more capable of solubilizing PAHs than a single surfactant [27]. In addition, the co-solubilization of binary PAH mixtures has also been studied [30,31,32,33,34]. For example, Sneha Singh et al. reported that anthracene (Ant) and Pyr, Pyr and fluorene had a synergistic solubilization effect in C_16_E_7_ surfactant micelles, while an inhibitory solubilization effect occurred for Ant and fluorine [30]. Moreover, synergistic solubilization effects were observed for Naphthalene and Pyr in polysorbate 80 (Tween 80), polyoxyethylene cetyl ether (Brij58), cetyltrimethylammonium bromide (CTAB) and sodium dodecyl benzenesulfonate (SDBS) micelles, but an inhibited solubilization effect occurred in polyethylene glycol octadecyl ether (Brij78) micelles [33]. Liang et al. reported that Phe and Pyr had a synergistic effect in sodium dodecyl sulfate (SDS) and tert-octyl phenoxy polyethylene ethoxy ethanol (TX100) single micelles and SDS-TX100 mixed micelles [34]. These studies indicate that the synergistic or inhibitory solubilization effect may occur between different PAHs or in different surfactant micelles. This is probably because the mechanism of the synergism/inhibition of the co-solubilization system is complicated.

In addition, in actual environment, PAHs mostly exist in the form of various mixtures. However, there is only a few studies paid attention to the co-solubilization of multiple PAH mixtures, such as ternary PAH mixture. Uzma Ashraf et al. found that the binary and ternary mixtures of Phe, Pyr and perylene could solubilize more PAHs in the shell region with dense surfactant micelles, thus increasing the core volume of micelles and enhancing the solubilization of PAHs [35]. It needs to mention that whether it is the co-solubilization of binary or ternary mixtures, all these above studies mainly investigated on the mutual influence of the solubility of PAHs in the presence of other PAHs. To our best knowledge, the total solubility and the relationship between the micellar environment and the overall solubilization of multi-PAHs have not been investigated.

Nuclear magnetic resonance (NMR) method can provide abundant qualitative and quantitative information about the solubility, solubilization sites, and the chemical environment of micelles and each of the solubilized PAHs at the molecular or atomic level. There are many studies reported about the solubilization of PAHs by NMR methods [19,30,32,36]. E. Takeuchi et al. found that naphthalene was solubilized in the palisade region of C_16_E_7_ micelles by ^1^H-NMR and rotating frame overhauser effect spectroscopy (ROESY) methods [30]. ^1^H-NMR spectroscopy showed that Phe, naphthalene and fluorene were solubilized in the palisade and core regions of TX100-SDS mixed micelles [32]. In this study, the co-solubilization of three PAHs of Phe, Ant and Pyr in Tween 80 and SDS were studied by ^1^H-NMR and 2D nuclear overhauser effect spectroscopy (NOESY) and ROESY methods. Relationships between the synergism/inhibition level and the solubilization sites and mechanisms, the total solubility and the chemical environment were explored and discussed, which is expected to provide research fundamentals for the practical applications of PAHs.

## 2. Results and Discussion

### 2.1. Solubilization of Phe, Ant and Pyr in Tween 80

#### 2.1.1. Apparent Solubility

**Scheme 1:**
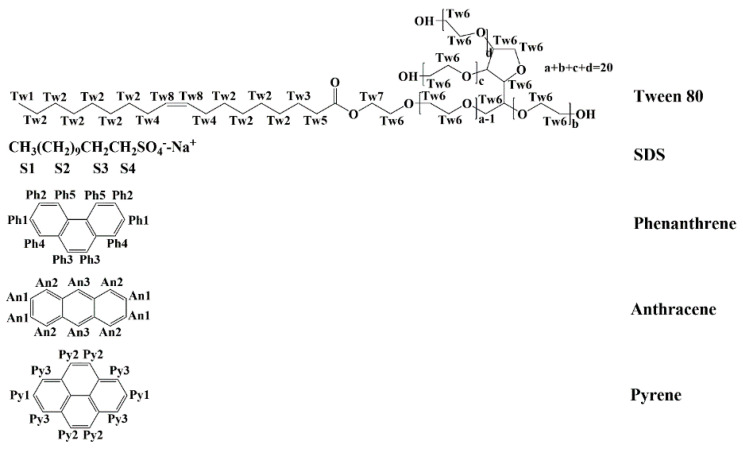
Chemical structures and proton numbering of surfactants and PAHs.

**Figure 1:**
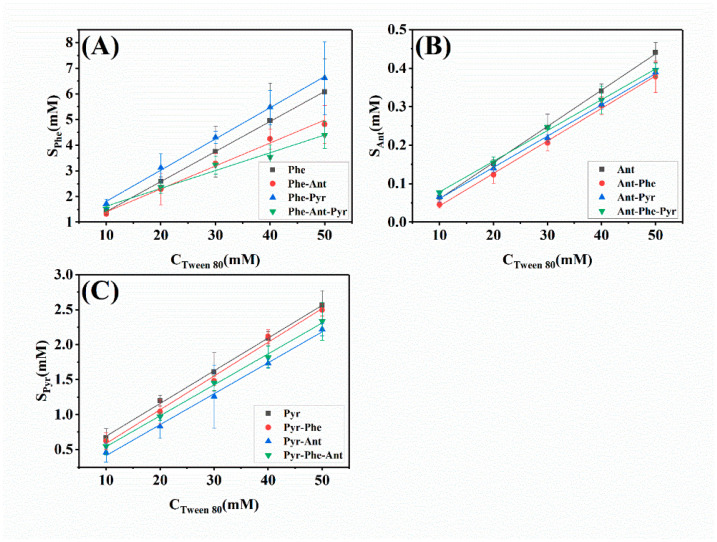
The apparent solubility (S) of Phe (**A**), Ant (**B**) and Pyr (**C**) in single, binary or ternary states in Tween 80 micellar solutions. The apparent solubility is shown as the mean and the standard deviation of three measurements.

Scheme 1 shows the chemical structures and proton numbering of surfactants and three PAHs of Phe, Ant and Pyr. The represented ^1^H-NMR spectra of ternary PAHs solubilized in 50 mM Tween 80 micellar solutions and pure ternary PAHs solubilized in chloroform and pure 50 mM Tween 80 in D_2_O as references are shown in Figure S1, Supplementary Materials. Based on the integral areas of resonances Ph5, An3 and Py2 relative to that of TSP with the known concentration of 2.82 mM, the actual apparent concentrations of three PAHs were obtained. The apparent solubility of each PAHs in single, binary and ternary solubilization systems as a function of the Tween 80 concentration are shown in Figure 1, Supplementary Materials. It can be found that all the solubilities of Phe, Ant and Pyr increase linearly with the Tween 80 concentration, whenever they are solubilized in single or combined systems. Molar solubilization ratio (*MSR*) is used to evaluate the solubilization potential of a surfactant, which is the molar concentration of the solubilized compound per mole of the surfactant concentration when the surfactant concentration is above CMC [37,38], expressed by:

(1) $$MSR=\frac{S-S_{CMC}}{C-\mathrm{CMC}}$$

where *S* is the apparent solubility of a solute at the surfactant concentration of *C*, *S_CMC_* is the apparent solubility of the solute at CMC. Usually, the slope of the solubility with respect to the surfactant concentration can be considered to be *MSR*. According to the linear fitting curves, *MSR* values of three PAHs in single, binary and ternary solubilization systems in Tween 80 solutions can be obtained and shown in Table 1. *MSR* values of single Phe, Ant and Pyr are 0.1170, 0.0094 and 0.0468, respectively. Accordingly, single Phe has the strongest solubilization ability, followed by single Pyr, and finally Ant, which are in accordance with the order of their aqueous solubility of 5.55 μM [39], 0.684 μM [40] and 0.268 μM [24]. The aqueous solubility is closely related to their structures. Phe and Ant are isomers; however, because three benzene rings of Ant are in a line, therefore Ant exhibits weaker polarity than Phe. For Pyr, there is one more benzene ring than Phe. Hence, its polarity is also weaker than that of Phe. The above single solubility results indicate that the solubility of PAHs in micellar solutions mainly depends on its aqueous solubility.

To compare the mutual effects of solutes in co-solubilization systems, the deviation ratio of *MSR* (R_ΔMSR_) can be used to evaluate the effects of synergistic (R_ΔMSR_ > 0) or inhibitory (R_ΔMSR_ < 0), which can be expressed by [32,33]:

(2) $$R_{\Delta MSR}\%=\frac{MSR_{mixture}-MSR_{single}}{MSR_{single}}\times 100\%$$

where MSR_mixture_ and MSR_single_ are the MSR values of solutes in the binary or ternary co-solubilization system and in the single solubilization system, respectively.

**Figure 2:**
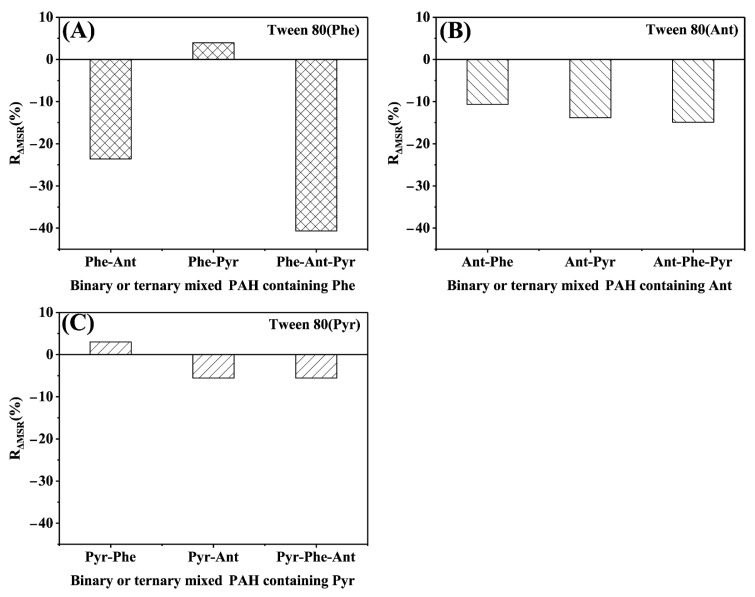
R_ΔMSR_ of Phe (**A**), Ant (**B**) and Pyr (**C**) in binary and ternary co-solubilization systems in Tween 80 micellar solutions.

Synergistic/inhibitory level (R_ΔMSR_) of Phe (A), Ant (B) and Pyr (C) in binary and ternary co-solubilization systems in Tween 80 solutions are shown in Figure 2. For Phe, inhibition is shown in binary Phe-Ant and ternary Phe-Ant-Pyr co-solubilization systems, while synergism occurs for Phe-Pyr. For Ant, slight inhibition is shown in all binary Phe-Ant, Ant-Pyr and ternary Phe-Ant-Pyr combination. For Pyr, inhibition is observed in the presence of Ant for both Ant-Pyr and Phe-Ant-Pyr, while synergism is shown for Phe-Pyr. It has been found that the more polarizable PAHs incline to solubilize in the palisade layer because of the π-electrons in the aromatic rings, and vice, the more hydrophobic ones would replace the others from the micelle core [33]. According to the single micellar solubility of three PAHs and the literature report, we speculate that Phe and Pyr are more likely solubilized in the palisade layer, and Ant is possibly in the micelle core. However, when Ant is co-solubilized, the decreased solubility for Phe and Pyr shows that a part of Ant is also probably located in the palisade layer. Meanwhile, the reduced solubility for Ant in Phe-Ant, Ant-Pyr, and Pyr-Ant-Phe suggests that some Phe, Pyr are also solubilized in the micellar core. Saumyen Guha et al. [41] found that: if PAHs compete with each other for the internal position of micelles, the solubility of one or more PAHs in the presence of other PAHs will be reduced. The solubility decrease of Phe, Pyr and Ant when they are co-solubilized is probably due to the competition for the micelle core and palisade layer. Moreover, it was also mentioned that the less hydrophobic PAHs solubilized in the palisade layer can reduce the interfacial tension and result in the increase of the micellar volume to solubilize more PAHs [33]. Therefore, the mutual synergistic solubilization effects for Phe-Pyr may be due to the increase of the Tween 80 micellar volume. In addition, for the decrease of the solubility of Phe and Pyr when Ant is co-solubilized, the presence of the hydrophobic Ant in the palisade layer may increase the interfacial tension, thus shrinking the micellar volume, which also results in the decrease of the solubility of Phe and Pyr to some extent.

#### 2.1.2. Solubilization Sites

**Figure 3:**
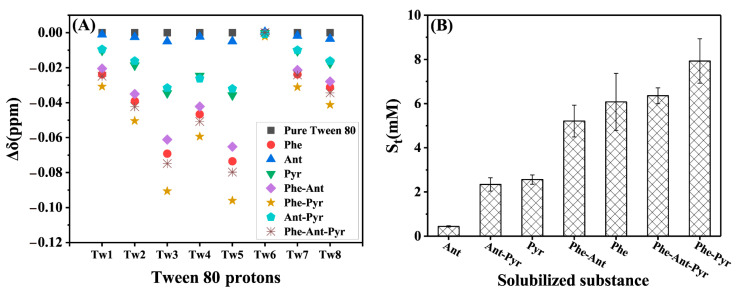
Chemical shift variations of Tween 80 protons (**A**) compared to those of pure Tween 80 and the total solubility (**B**) in different solubilization systems at the same Tween 80 concentration of 50 mM.

Chemical shifts are sensitive to the chemical environment changes of nucleus. Chemical shift changes of surfactants and solutes can provide abundant information about solubilization, such as the solubilization site, the solubilization effects on micellar properties, and even the solubility. Proton chemical shifts for Tween 80 with and without the solubilization of PAHs are shown in Table 2. To visualize it, the chemical shift changes of all Tween 80 protons in single, binary and ternary systems with different PAHs solubilized compared with those of pure Tween 80 at the same concentration of 50 mM are shown in Figure 3A. Compared to those of pure Tween 80, with the addition of Phe in Tween 80, chemical shifts of all the resonances except Tw6 change significantly (at least 0.002 ppm). As we know, Tw6 is located at the hydrophilic head region that is always surrounded by vast water molecules. Hence, its chemical shift is rarely changed. However, interestingly, it can be seen from Figure S2 that after the solubilization of Phe, a small peak (known as Tw6′) at the right bottom of Tw6 splits out and appears at a higher field than Tw6. Since Tw6 is composed of nearly 20 ethoxy group, we deduce that some of the bulky ethoxy group protons of Tw6 interact with the solubilized PAHs, which results in the split of Tw6 and appearance of Tw6′. The chemical shift changes of these Tween 80 protons after the addition of Phe show that Phe is solubilized not only in the hydrophobic micellar core but also in the palisade layer and the shielding effects of the π-electrons in the aromatic rings of Phe on these Tween 80 protons result in their chemical shifts to the upfield. This is consistent with the speculation in the discussion about the synergistic/inhibitory effects in the co-solubilization systems. Furthermore, compared with the protons Tw1 and Tw2 that comprise the hydrophobic core region, shift changes of Tw3 and Tw5 are the most significant, which indicates that more Phe partitioned in the palisade layer compared to the core region of Tween 80 micelles. Compared to Phe, although Pyr and Ant experience a smaller and subtle chemical shift changes, respectively, the chemical shift changes also follow the same pattern as that of the system of Phe. This implies that three PAHs may have similar solubilization sites, which might be because they all only have similar hydrophobic fused ring structures. Then, the similar molecular structure results in the similar interaction patterns between three PAHs and Tween 80, which in turn leads to similar solubilization sites. Likewise, the similar chemical shift change pattern can also be found in the co-solubilization systems of all binary and ternary mixtures of these three PAHs, such as shift changes of Tw3 and Tw5 are still the most significant, suggesting that the co-solubilization does not significantly change their solubilization modes and sites in single states.

#### 2.1.3. The Total Solubility and Its Characterization by Chemical Shift Changes

The assessment of the total solubility (S_t_) of PAHs is also important in practical application, because PAHs tend to co-exist in the environment. Therefore, the S_t_ of multi-solutes can reflect the actual and global solubility results. S_t_ in a mixed PAHs system is equal to the sum of the apparent solubility of each PAHs, and S_t_ in a single PAH system is the apparent solubility of the certain PAH. It has been found that the solubility of three single solutes follow the order of Phe > Pyr > Ant. Interestingly, the chemical shift changes follow the same order as the solubility, which means that when the most soluble Phe is solubilized, chemical shifts of Tween 80 protons vary the most, and vice versa. To understand this phenomenon, we proposed a hypothesis that the change of chemical shift is related to the solubility of PAHs. Under the condition of the same surfactant concentration *C*, the chemical shift of a certain proton group of the surfactant in pure solutions is set as *δ*_0_. When the PAH is solubilized, assuming that a PAH molecule interacts with *n* surfactant molecules in the micelles, and the chemical shift change of a certain proton group of these *n* surfactant molecules is set as Δ*δ*, thus, the chemical shift of these surfactant molecules (interacting sites) equals to Δ*δ + δ*_0_, while the chemical shift of the proton group of those surfactant molecules without interactions with PAHs (non-interacting sites) is still *δ*_0_. If the solubility of PAH is *S*, the *δ_obs_* of the proton group of all surfactant molecules is the average of chemical shift of interacting and non-interacting sites shown in the following Ep. 3, Based on this relationship between chemical shift and solubility, we can find that *δ_obs_* is proportional to the solubility. This implies that the change of chemical shift might be used as an indicator for the solubility.

(3) $$\delta_{obs}=\frac{n}{C}\cdot \Delta \delta \cdot S+\delta_{0}$$

Then, we further explored the relationship between the solubility and chemical shift change in co-solubilization systems. The S_t_ of each system with PAHs solubilized in single, binary and ternary states are shown in Figure 3B. As we have mentioned, the chemical shift changes of Tween 80 in different solubilization systems follow the similar patterns shown in Figure 3A, but to a different degree. It follows the order of Phe-Pyr > Phe-Ant-Pyr > Phe> Phe-Ant > Pyr > Ant-Pyr > Ant, interestingly, which is also consistent with the order of the total solubility in Figure 3B, but not the single solubility of any PAHs. This indicates that the chemical environment change of Tween 80 micelles depends on S_t_ of all solubilized PAH solutes. Moreover, chemical shift variations of PAH protons shown in Figure S3 can also reflect the S_t_ level. When the S_t_ is high, the chemical shift of PAHs protons varies the most, vice versa. These can be interpreted that the more the total PAHs solubilized, the more hydrophobic the Tween 80 micelles, and the more hydrophobic the chemical environment in which PAHs are solubilized.

In Figure 3B, the S_t_ of the systems including Phe ranks highly due to the highest intrinsic solubility of Phe among these three solutes. There into, compared to the single Phe, when Pyr is co-existed, the total solubility is higher whenever in binary or ternary systems. However, the presence of the inhibitory Ant decreases the S_t_ of the binary Phe-Ant, so does another binary Ant-Pyr. Although Ant is inhibitory to both Phe and Pyr, the S_t_ in the ternary system is still higher than that of the single Phe, which suggests a good solubilization effect can be expected when these three PAHs are co-existed in the environment. In conclusion, first, the S_t_ mainly depends on the solubility of the most soluble solute. Second, synergism promotes the S_t_, vice versa. Last, the S_t_ also depends on the balance between the synergistic/inhibitory effects of the solutes.

#### 2.1.4. Average Total Solubility and Its Characterization by Chemical Shift Changes

**Figure 4:**
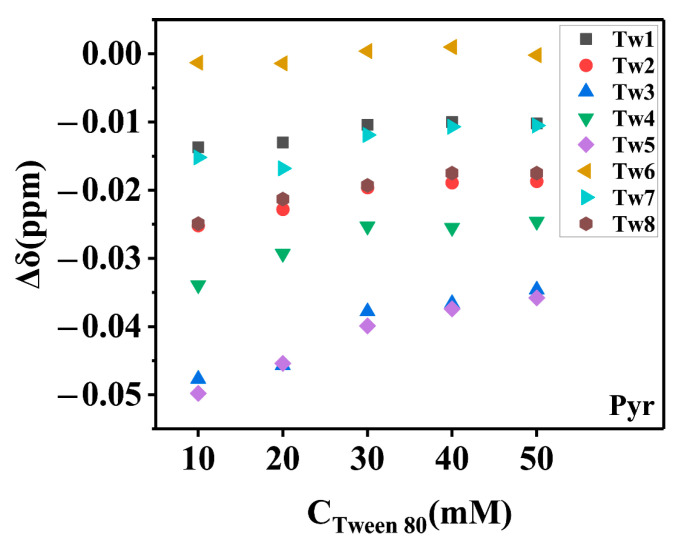
Relative chemical shifts (∆δ) of Tween 80 protons in the single Pyr solubilization system at Tween 80 concentrations from 10 to 50 mM compared to those of the pure Tween 80 solution at the same concentration.

**Figure 5:**
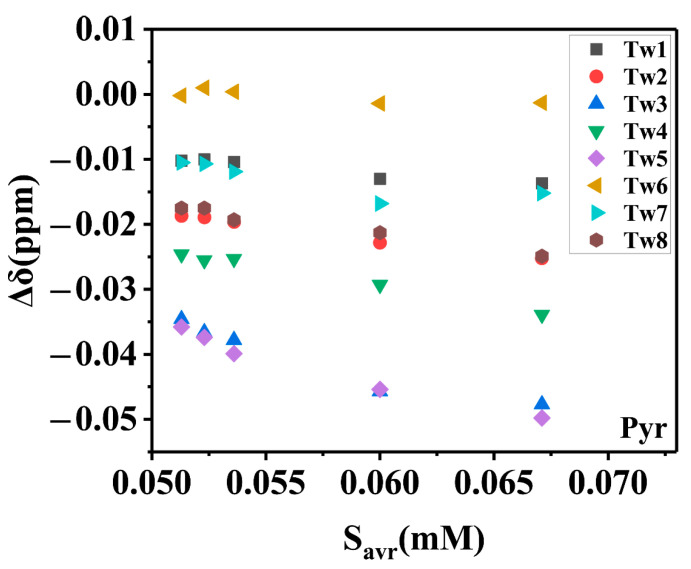
Relative chemical shifts (∆δ) of Tween 80 protons as a function of S_avr_ in the single Pyr system at Tween 80 concentrations from 10 to 50 mM compared to those of the pure Tween 80 solution at the same concentration.

Furthermore, the relationship between the chemical shift and solubility of the same PAHs systems at different Tween 80 concentrations was also explored. Relative chemical shifts of Tween 80 protons in the single Pyr solubilization system at Tween 80 concentrations from 10 to 50 mM compared to those of the pure Tween 80 solution at the same concentration as an example are shown in Figure 4. We can find that as the solubility of Pyr increases with the increase of Tween 80 concentration, a different phenomenon shows that except for Tw6, relative chemical shifts of nearly all Tween 80 protons move to the downfield. This can also be observed from the other PAHs solubilization systems in Figure S4. This is against the conclusion obtained from the above experimental results that the higher the S_t_, the upfield the chemical shift. After careful analysis, we find that although the S_t_ increases with the increase of Tween 80 concentration, the average total solubility (S_avr_, the solubility per mole of Tween 80) of Pyr decreases. As S_avr_ increases, relative chemical shifts of nearly all Tween 80 protons move to the upfield as shown in Figure 5. This is consistent with the conclusion that the more PAHs solubilized, the more hydrophobic the Tween 80 micelles, and the upfield the chemical shift. Similar results can also be obtained in other PAHs solubilization systems as shown in Figure S5.

#### 2.1.5. 2D ROESY

**Figure 6:**
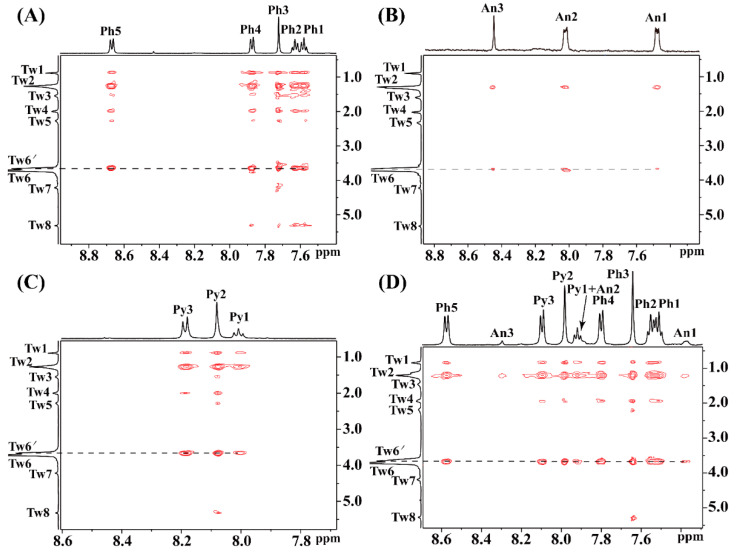
Partial ROESY spectra of single Phe (**A**), Ant (**B**), Pyr (**C**) and ternary Phe-Ant-Pyr systems (**D**) at the Tween 80 concentration of 50 mM with the mixing time of 0.2 s.

To verify the solubilization sites of PAHs in Tween 80 micelles deduced from the chemical shift changes in ^1^H-NMR spectra, selected regions of ROESY spectra of single and ternary of Phe, Ant and Pyr solubilized at the Tween 80 concentration of 50 mM with the mixing time of 0.2 s shown in Figure 6 were obtained, respectively. In the system of single Phe (Figure 6A), there are cross peaks between all the protons of Phe and Tw1, Tw2, Tw3, Tw4, Tw5, Tw6′, and cross peaks between Tw8 and the four proton groups of Phe, and Tw7-Ph3. These prove that the solubilization sites of Phe in Tween 80 micelles are in both the hydrophobic core and the palisade layer. However, for Ant, all the protons of Ant only have cross peaks with Tw2 and Tw6, but no cross peaks with Tw3 and Tw5, while their chemical shifts vary the most (Figure 6B). We speculate that unlike Tw2 and Tw6, which have many protons, the protons of Tw3 and Tw5 are very few. Moreover, the solubility of Ant is also very low. Therefore, it is hard to produce visible cross peaks. However, it can also be concluded that Ant is also solubilized in the palisade and hydrophobic core of Tween 80 micelles. For Pyr, there are cross peaks between all the protons of Pyr and Tw1, Tw2 and Tw6′, and cross peaks between Py2 and Tw3, Tw4, Tw5 and Tw8, and Tw4-Py3, which proves that Pyr also has the similar solubilization sites as Phe and Ant (Figure 6C).

When Phe, Ant and Pyr are co-solubilized in Tween 80 micelles (Figure 6D), for Phe, some cross peaks are reduced, such as cross peaks between Tw3 and all Phe protons, Tw5 and Tw8 and some Phe protons, Tw7-Ph3 are disappeared. For Ant, cross peaks between An2, An3 and Tw6 are disappeared. For Pyr, cross peaks between Py2 and Tw3, Tw5, Tw8 are disappeared. The disappearance of the cross peaks between Phe, Ant and Pyr protons and Tween 80 protons further confirm the competitive solubilization sites among Phe, Ant and Pyr, which leads to the inhibitory effects of Phe, Ant and Pyr in the ternary Phe-Ant-Pyr mixture. In addition, the entire ROESY spectrum of the ternary mixture is also shown in Figure S6. From Figure S6, we can find there are cross peaks between water and Tw6, which directly proves that Tw6 is extensively surrounded by water. It is worth noting that there are some changes in cross peaks compared with the single systems; however, the mainly strong cross peaks, such as PAHs and Tw2, Tw6′, almost are unchanged, which supports the conclusion obtained from chemical shift changes that the co-solubilization does not significantly change their solubilization sites in single states.

It needs to mention that cross peaks are always supposed to be shown up as pairs. However, for the cross peaks between PAHs and Tween 80, they only show up in the upper half of the figure, which are with Tw2 and Tw6 as sources. In the lower part of the figure, the symmetric cross peaks with PAH protons as sources are not shown up. One possible reason is that the number of solubilized PAHs is too small to produce visible cross peaks. Another is that strong resonances such as Tw2 and Tw6 generate strong t1 noises along the *F_1_* axis, which possibly affect the observation of the cross peaks in the lower part of the figure.

### 2.2. Solubilization of Phe, Ant and Pyr in SDS

#### 2.2.1. Apparent Solubility

**Figure 7:**
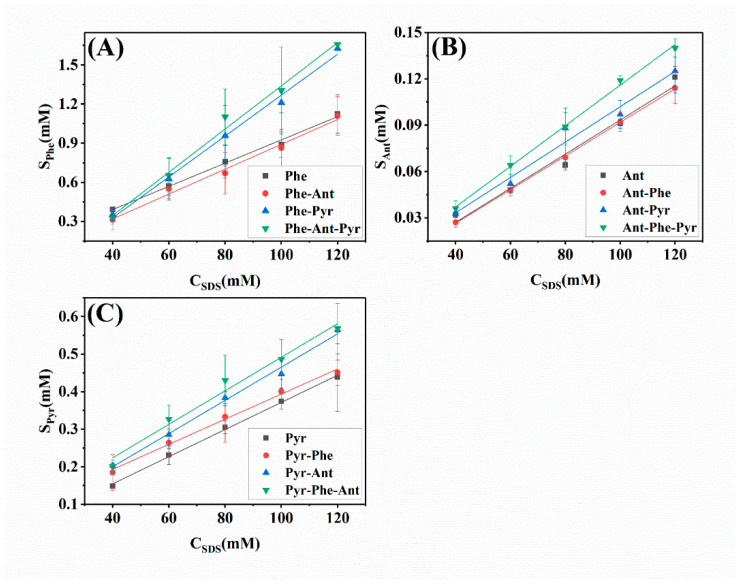
The solubility of Phe (**A**), Ant (**B**) and Pyr (**C**) in single, binary or ternary solubilization systems in SDS micellar solution.

The represented ^1^H-NMR spectra of three PAHs solubilized in 120 mM SDS micellar solutions and pure three PAHs solubilized in chloroform and 120 mM pure SDS in D_2_O as references are shown in Figure S7. The variation of apparent solubility of PAHs in single, binary and ternary combinations with the concentration of SDS are shown in Figure 7. It can be found that all the solubility of Phe, Ant and Pyr also increases linearly with SDS concentration. Similarly, *MSR* values of three PAHs in SDS solutions can be obtained, as shown in Table 3. *MSR* values of single Phe, Ant and Pyr in SDS are 0.0089, 0.0011 and 0.0036, respectively. As the same as in Tween 80, single Phe has the strongest solubilization ability, followed by single Pyr, and finally Ant.

**Figure 8:**
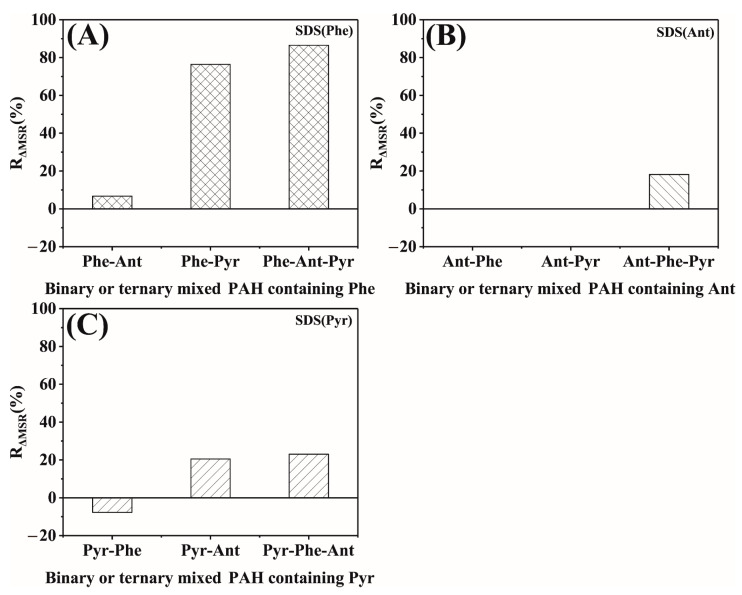
R_ΔMSR_ of Phe (**A**), Ant (**B**) and Pyr (**C**) in binary and ternary co-solubilization systems in SDS micellar solutions.

R_ΔMSR_ of binary and ternary combinations of Phe, Ant and Pyr compared to single Phe, Ant and Pyr are shown in Figure 8. For Phe, synergism is shown in all binary Phe-Ant, Phe-Pyr and ternary Phe-Ant-Pyr. Especially for the binary Phe-Pyr and ternary Phe-Ant-Pyr, the solubility of Phe dramatically increases by 76.4% and 86.5%, respectively. For Ant, the presence of neither Phe nor Pyr contributes to the solubility increase of Ant. However, in the ternary system of Phe-Ant-Pyr, it shows slight synergism. For Pyr, in both binary Pyr-Ant and ternary Phe-Ant-Pyr systems, they show synergism. However, the solubility of Pyr is suppressed due to the presence of Phe. Liang et al. [34]. found that water molecules can permeate into the hydrophobic region of SDS micelles, which is also found in our study (See Section 2.2.5). This indicates that the structure of SDS micelles is relatively loose due to the repulsion among ionic heads. Therefore, we speculate that compared to Tween 80 micelles, SDS micelles with the loose micellar structure are easy to enlarge to solubilize more PAH molecules, which results in the synergistic effects. However, the decrease of Pyr solubility is probably due to the remarkable increase of Phe, if the micellar solubilization region is limited. For Ant, no significant increase in solubility may be resulted from the extremely hydrophobic property. The solubilization of Ant may mainly depends on the limited solubilization sites on the hydrophobic chain of the SDS surfactant.

#### 2.2.2. Solubilization Sites

**Figure 9:**
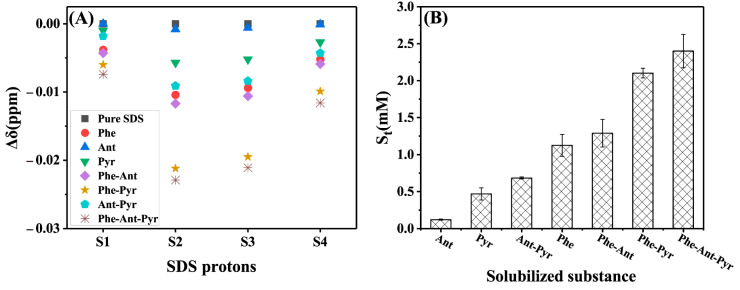
Chemical shift variations of SDS protons (**A**) compared to those of pure SDS and the total solubility (**B**) in different solubilization systems at the same SDS concentration of 120 mM.

Proton chemical shifts of SDS with and without the solubilization of PAHs are shown in Table 4. Chemical shift changes of all SDS protons compared with those of pure SDS at the same concentration of 120 mM are shown in Figure 9A. It can be found that chemical shifts of nearly all protons of SDS also move to an upfield to some extent after the solubilization of the hydrophobic PAHs. In addition, shifts of protons S2 and S3 in the hydrophobic methylene chain change greater than those of S4 in the palisade layer and the terminal protons S1, suggesting that PAHs are mainly solubilized in the hydrophobic core, rather than largely in the palisade layer as with Tween 80 micelles. The repulsive force between π-electrons of PAHs and the negative charge of the head group of SDS probably lead to the less solubilization of PAHs in the palisade layer of SDS micelles.

Similar to Tween 80, the chemical shift changes of SDS protons in different solubilization systems of PAHs are similar, which suggests that although the main solubilization sites of PAHs in SDS and Tween 80 micelles are different, due to the similar molecular structures, three PAHs also have similar solubilization sites in SDS micelles. This property can also be found that the similar chemical change patterns of Naphthalene and Pyr due to the similar fused ring structures are also shown in the single or co-solubilization in nonionic Brij series or Tween 80 and anionic SDBS micelles [33]. However, in cationic surfactant CTAB micelles, it does not follow the same rule due to the electrostatic interaction between the π-electrons of PAHs and the positive ion heads of CTAB. Naphthalene and Pyr compete to interact with the positive charges along with the co-solubilization, resulting the change of their original interaction modes in single states [33].

#### 2.2.3. The Total Solubility and Its Characterization by Chemical Shift Changes

The S_t_ of each system with PAHs solubilized in single, binary and ternary states in SDS are shown in Figure 9B. Likewise, the S_t_ of PAHs and chemical shift changes of SDS protons follow the same order of Phe-Ant-Pyr > Phe-Pyr > Phe-Ant > Phe > Ant-Pyr > Pyr > Ant. The variation in chemical shift of PAHs protons also follows the same order. (Figure S8). Therefore, these experimental results also support the conclusion deduced from Tween 80 solubilization systems that the more PAHs solubilized, the more hydrophobic SDS micelles, and the more hydrophobic chemical environment in which PAHs are located.

In Figure 9B, the S_t_ of the systems including Phe also ranks highly as the same as in Tween 80 solutions. However, the S_t_ order of PAHs in SDS is different from that in Tween 80. In SDS, because both Ant and Pyr have synergistic solubilization effects on Phe, therefore, S_t_ of ternary Phe-Ant-Pyr is the highest, followed by binary Phe-Pyr and Phe-Ant. Similarly, the total solubility of binary Ant-Pyr system is higher than that of single Pyr system. In short, the conclusion in SDS is the same as that in Tween 80. The S_t_ of the ternary PAHs mainly depends on the solubility of the most soluble solute and partially the balance between the synergistic and inhibitory effects of the solutes.

#### 2.2.4. Average Total Solubility and Its Characterization by Chemical Shift Changes

**Figure 10:**
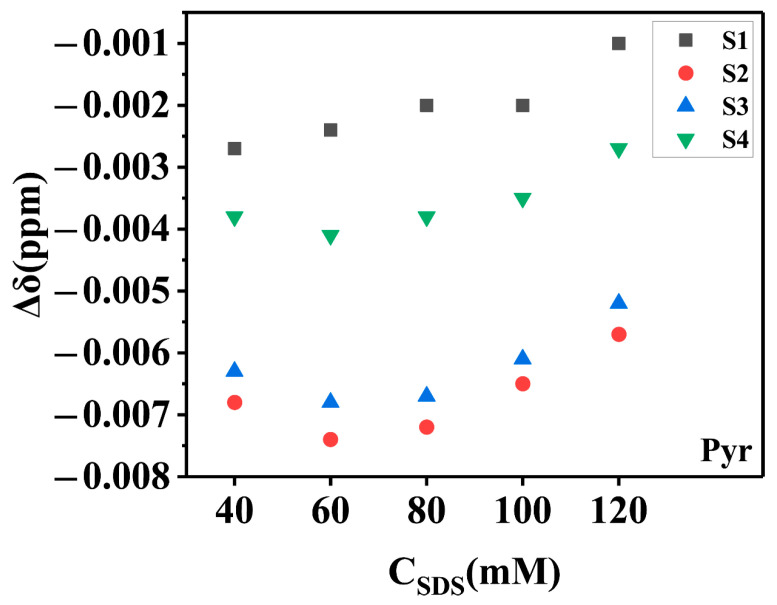
Relative chemical shifts (∆δ) of SDS protons in the single Pyr solubilization system at SDS concentrations from 40 to 120 mM compared to those of the pure SDS solution at the same concentration.

Furthermore, the relationship between the chemical shift and solubility of the same PAHs systems at different SDS concentrations was also explored. Relative chemical shifts of SDS protons in the single Pyr solubilization system at SDS concentrations from 40 to 120 mM compared to those in the pure SDS solution at the same concentration as an example are shown in Figure 10. It can be found that as the S_t_ of Pyr increases with the increase of SDS concentration, relative chemical shifts of nearly all SDS protons move to the downfield, which is against with our conclusion that the higher the S_t_, the upfield the chemical shift. Relative chemical shifts of SDS protons in other different PAHs solubilization systems can be obtained and shown in Figure S9. It can be found that Phe, Phe-Ant and Ant-Pyr systems have similar results with the Pyr system. However, for Phe-Pyr and Phe-Ant-Pyr systems, relative chemical shifts of nearly all SDS protons move to the upfield, and for the Ant system, relative chemical shifts of nearly all SDS protons move to the downfield first and then move to the upfield.

**Figure 11:**
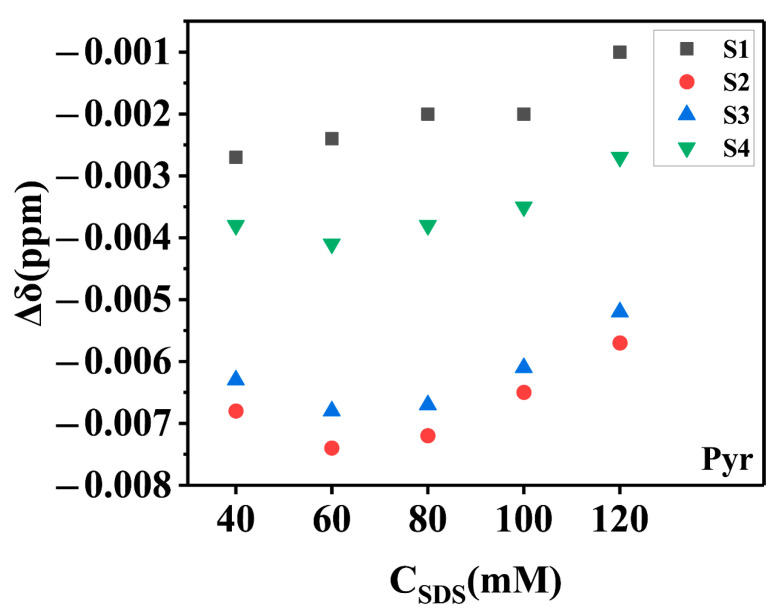
Relative chemical shifts (∆δ) of SDS protons as a function of S_avr_ in the single Pyr system at SDS concentrations from 40 to 120 mM compared to those of the pure SDS solution at the same concentration.

Confronted with these different total solubility results, we also further analyzed the relationship between the relative chemical shift and S_avr_. Relative chemical shifts of SDS protons as a function of S_avr_ in the single Pyr system at SDS concentrations from 40 to 120 mM as an example are shown in Figure 11. It can be found that with the increase of S_avr_, the relative chemical shifts of nearly all SDS protons move to the upfield. Similar results can also be obtained in other PAHs solubilization systems as shown in Figure S10. This is consistent with the conclusion obtained in Tween 80 that the more PAHs solubilized, the more hydrophobic the SDS micelles, and the upfield the chemical shift.

#### 2.2.5. 2D NOESY

**Figure 12:**
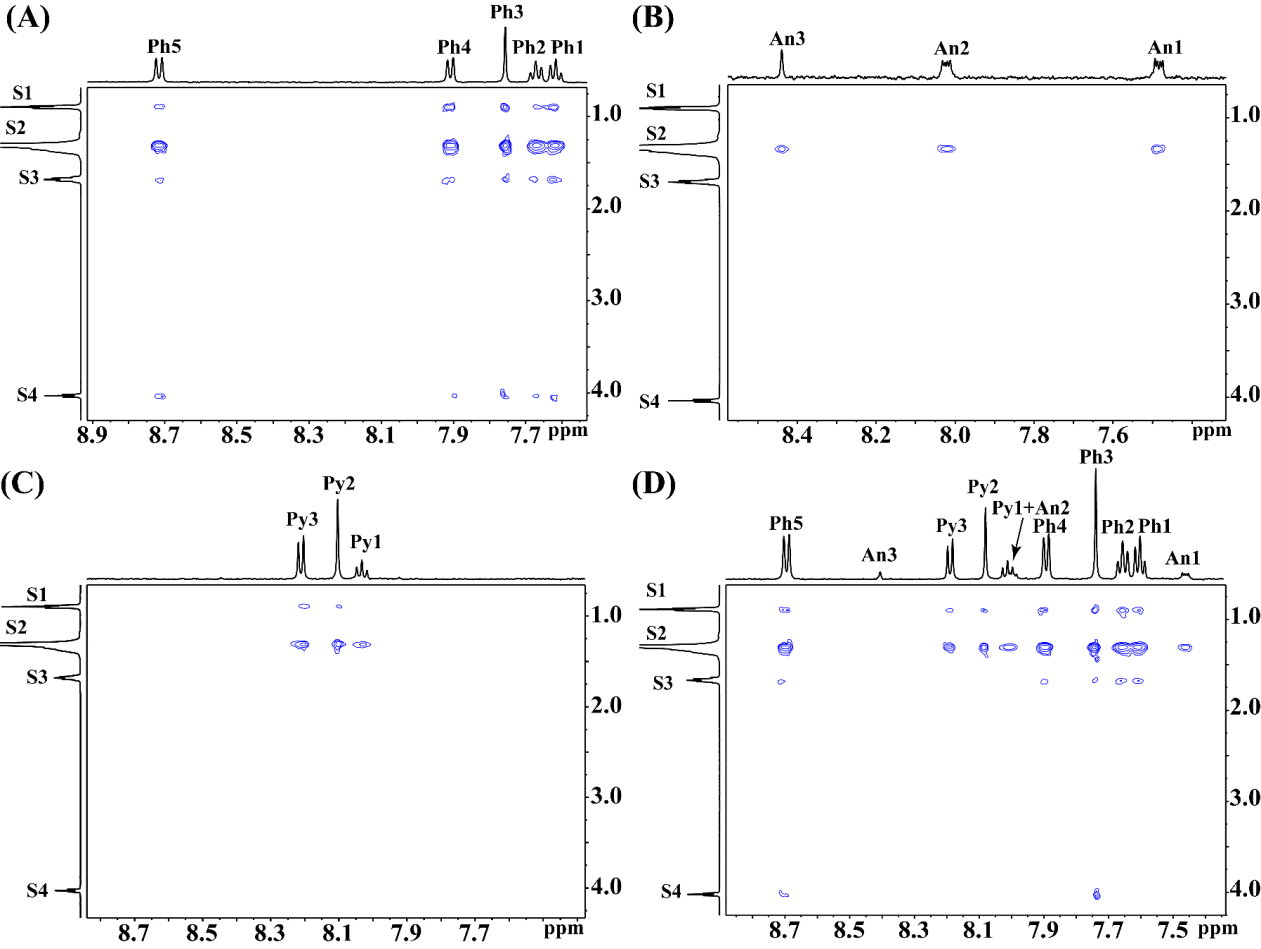
Partial NOESY spectra of single Phe (**A**), Ant (**B**), Pyr (**C**) and ternary Phe-Ant-Pyr systems (**D**) solubilized by 120 mM SDS micelles with the mixing time of 1 s.

In the whole NOESY spectrum of the ternary PAHs shown in Figure S11, most of the cross peaks are anti-phase with the diagonal peak (called negative cross peaks, vice versa). However, there is a pair of positive cross peaks from S4 and S2, which indicates that SDS micelles form. The negative cross peaks among SDS and PAHs protons themselves and the cross peaks between PAHs and SDS, demonstrate that three PAHs are all solubilized in them and the molecular motion of SDS and solubilized PAHs is relatively free. In addition, except for S1, there are cross peaks between water and all SDS protons, which shows that water penetrates the SDS micelles. This is in good accordance with the literature report [34] and proves that the SDS micellar structure is loose. In the systems of single Phe, Ant and Pyr, there are strong cross peaks between all proton groups of PAHs and S2 protons of SDS (Figure 12A–C), proving that PAHs are mainly solubilized in the hydrophobic cores of SDS micelles. Moreover, there are cross peaks between all Phe protons and S4 and S3, suggesting there are a few Phe solubilized in the palisade layer of SDS micelles. From the chemical shift change of SDS protons, we deduced that three PAHs have similar solubilization sites, mainly in the hydrophobic cores and partially in the palisade layer. However, the cross peaks between Pyr and Ant and the palisade protons S4 and S3 are absent, which is probably due to the low solubility of Pyr and Ant. When Phe, Ant and Pyr are co-solubilized (Figure 12D), for Pyr and Ant, cross peaks are basically the same as those in single states, showing the solubilization sites does not change significantly. For Phe, most cross peaks are the same as those in single state. However, some cross peaks between Phe protons and S4 disappear, showing that less Phe molecules are solubilized in the palisade layer. We deduce that more Phe is solubilized in the micellar core due to the electronic repulsion between the head group and π-electrons of benzene rings as the solubility increases in ternary system. Similarly, for the cross peaks between PAHs and SDS, in the lower part of the figure, it is also difficult to find the other half of the cross-peak pairs.

### 2.3. General Mechanisms for the Relationship between the Chemical Shift Change and the Total Solubility

We have concluded that the similar solubilization sites depend on the similar molecular structures of PAHs when they are solubilized in single states. However, when multi-solutes co-exist in micellar solutions, based on the experiments from this study and the literature [33], the similar solubilization sites not only depend on the similar molecular structure of solutes, but also the interaction between PAHs and the surfactant micelles. In this study, Tween 80 is a kind of nonionic surfactant, and the hydrophobic interaction is the main interaction between PAHs and Tween 80, which is the same when they are in single or mixed states. For SDS micelles, although except the hydrophobic interaction, there is the repulsive interaction between the π-electrons of PAHs and the negative head group of SDS micelles, it is favor to the hydrophobic interaction, which means that there is only one type of interaction between these two micelles and PAHs. Therefore, the interaction mode between PAHs and SDS micelles in binary and ternary combinations are the same as PAHs in single states. However, if the surfactant is cationic, there is an electrostatic attractive interaction between the π-electrons of PAHs and the positive head group of cationic surfactants, which is an opposite interaction with the hydrophobic interaction between PAHs and the hydrophobic chains. Since there is not only one type of interaction, when PAHs are co-solubilized, the solubility and solubilization sites of PAHs depend on the balance between the electrostatic attractive interaction in the palisade layer and the hydrophobic interaction in the micellar core, and the competition among different PAHs for solubilization. These will lead to the change in the content and proportion of PAHs in palisade layer and the micellar core and further result in the different chemical shift change pattern as that in single state. In this case, we cannot use chemical shift to evaluate the S_t_ of co-solubilization systems.

## 3. Materials and Methods

### 3.1. Materials

Nonionic surfactant Tween 80 (MW = 1310, 99%) and anionic surfactant SDS (MW = 288.38, 99%) were purchased from Sigma-Aldrich, Shanghai, China. Deuterium oxide (D_2_O, 99.9%) was purchased from Qingdao Tenglong Weibo Technology, Qingdao, China. Phenanthrene (MW = 178.23, 97%) and anthracene (MW = 178.23, 99%) were purchased from Shanghai Macklin Biochemical Co., Ltd., Shanghai, China. Pyrene (MW = 202.25, 99%) was purchased from Shanghai Aladdin Biochemical Technology Co., Ltd., Shanghai, China. All reagents were used directly without further purification. First, a series of Tween 80 pure solutions at concentrations of 10, 20, 30, 40 and 50 mM and SDS pure solutions at concentrations of 40, 60, 80, 100 and 120 mM were prepared in D_2_O, respectively. These concentrations were set higher than their CMCs [32,42]. The solubilization experiment was processed as follows: first, 8 equal 800 μL surfactant solutions at a certain surfactant concentration were injected into eight of 1.5 mL centrifuge cuitubes, respectively. One of them without the addition of PAHs was used as the blank control. Then, excess PAHs (20 mg of each PAH) were added into the remaining 7 surfactant solutions in the single (Phe, Ant, and Pyr), binary (Phe-Ant, Phe-Pyr, and Ant-Pyr) or ternary (Phe-Ant-Pyr), respectively. Next, the surfactant solutions with PAHs solubilized were equilibrated on a reciprocating water bath oscillator at 200 rpm at 25 °C for 48 h. After equilibration, the above solutions were centrifuged at 10,000 rpm for 10 min. Finally, each of 600 μL of supernatant was transferred into 5 mm NMR tubes for further detection.

### 3.2. NMR Experiments

All NMR experiments were performed at 25 °C using a 500 MHz Agilent ProPulse NMR spectrometer (Agilent Technologies, Santa Clara, CA, USA) equipped with a 5 mm XYZ indirect detection probe and z-direction gradient coils with a maximum nominal gradient strength of 60 G cm^−1^. The pulse excitation angle of 30 ° and the relaxation delay time of 5 s in ^1^H-NMR experiments were chosen to assure the complete recovery of the magnetization and the accurate quantification. The Sodium Trimethylsilylpropionate (TSP) aqueous solution in the capillary was used as an external reference for the frequency and concentration calibration. The chemical shift of TSP was set to *δ*0 and the concentration of TSP used in all experiments was 2.82 mM. Based on the resonance integral area of PAHs relative to that of TSP, we calculated their actual apparent concentrations. A total of 16 accumulations were used in 2D NOESY experiments, 1 s was chosen as mixing time. A total of 4k and 256 sampling data points were used for the direct dimension (*t_2_*) and indirect dimension (*t_1_*), respectively, and the Fourier transform data point array used after zero filling was 8 k (*F_2_*) × 1k (*F_1_*). Due to the high growth rate of rotating frame overhauser effect (ROE) than that of NOE, ROESY experiments for Tween 80 systems were conducted instead of NOESY to avoid the possible spin diffusion effect in NOESY with the long mixing time. The parameters of the 2D ROESY experiments were the same as the NOESY experiments except that the mixing time was 0.2 s. MestReNova software (version 14.0.0, Mestrelab Research, Santiago de Compostella, Spain) was used to process all NMR data.

## 4. Conclusions

In this study, the co-solubilization of three PAHs in Tween 80 and SDS are studied by NMR spectroscopy. The experimental results show that in two micellar solutions, the different synergic/inhibitory effects of PAHs are shown when they are co-solubilized. In Tween 80, for Phe, Ant and Pyr, the solubility is inhibited in all binary and ternary systems except the mutual synergistic solubilization of Phe and Pyr in the binary Phe-Pyr system. In SDS, except in the binary Phe-Pyr, the solubilization of Pyr is slightly inhibited, and Phe and Pyr have no effect on the solubilization of Ant, a synergistic solubilization effect is shown in most solubilization systems. These are mainly resulted from the different co-solubilization mechanisms. In Tween 80, three PAHs are solubilized in both palisade layer and micellar core. The more hydrophobic Ant leads to the inhibition of the solubility of Phe and Pyr. For SDS, the loose structure of SDS micelles may be the main reason for the synergism. Nevertheless, Phe, Ant and Pyr all have similar solubilization sites whenever in Tween 80 and SDS systems due to the similar structure of these three PAHs. Furthermore, in binary and ternary PAHs systems, compared to those in single state, their solubilization sites and the resulting chemical shift change pattern do not change significantly. This is because there is only one type of interaction between these two micelles and PAHs.

In addition, we also find that the total solubility order in different solubilization systems is consistent with the order of the chemical shift variations of both surfactants and PAHs. In this case, chemical shift can be used as an indicator of the S_t_. Furthermore, we also evaluate the total solubility of PAHs in Tween 80 and SDS micelles and conclude that the S_t_ mainly depends on the solubility of the most soluble solute, and the balance of the synergistic/inhibitory effects of the solutes. Due to the relatively high total solubility, a good overall solubilization effect can be expected when these three PAHs are co-existed in the environment. All in all, based on ^1^H-NMR, ROESY and NOESY, information about the solubilization sites of the three PAHs in Tween 80 and SDS, changes in the chemical environment of micelles, the solubility and its relationship with chemical shifts, and further the solubilization mechanisms are obtained, which can help people better understand the co-solubilization of PAHs.

## Figures and Tables

**Table 1:** *MSR* values of Phe, Ant and Pyr in single, binary or ternary solubilization systems in Tween 80 micellar solutions.

Surfactant	MSR	MSR	MSR	MSR
Tween 80	Phe			
	Phe (single)	Phe-Ant	Phe-Pyr	Phe-Ant-Pyr
	0.1170	0.0894	0.1216	0.0694
	Ant			
	Ant (single)	Ant-Phe	Ant-Pyr	Ant-Phe-Pyr
	0.0094	0.0084	0.0081	0.0080
	Pyr			
	Pyr (single)	Pyr-Phe	Pyr-Ant	Pyr-Ant-Phe
	0.0468	0.0482	0.0442	0.0442

**Table 2:** Proton chemical shifts (ppm) for Tween 80 with and without the solubilization of different PAHs at the Tween 80 concentration of 50 mM.

Segment	Pure	Phe	Ant	Pyr	Phe-Ant	Phe-Pyr	Ant-Pyr	Phe-Ant-Pyr
Tw1	0.9046	0.8837	0.9035	0.8944	0.8806	0.8756	0.8951	0.8797
Tw2	1.3033	1.2692	1.3008	1.2846	1.2639	1.2552	1.2871	1.2610
Tw3	1.6087	1.5493	1.6037	1.5741	1.5416	1.5239	1.5771	1.5339
Tw4	2.0411	1.9992	2.0388	2.0165	1.9944	1.9842	2.0149	1.9903
Tw5	2.3426	2.2788	2.3376	2.3068	2.2710	2.2535	2.3105	2.2629
Tw6	3.7172	3.7170	3.7176	3.7170	3.7156	3.7157	3.7164	3.7173
Tw7	4.2301	4.2108	4.2283	4.2196	4.2065	4.2020	4.2201	4.2059
Tw8	5.3435	5.3153	5.3400	5.3260	5.3118	5.3041	5.3273	5.3091

**Table 3:** *MSR* values of Phe, Ant and Pyr in single, binary or ternary solubilization systems in SDS micellar solutions.

Surfactant	MSR	MSR	MSR	MSR
SDS	Phe			
	Phe (single)	Phe-Ant	Phe-Pyr	Phe-Ant-Pyr
	0.0089	0.0095	0.0157	0.0166
	Ant			
	Ant (single)	Ant-Phe	Ant-Pyr	Ant-Phe-Pyr
	0.0011	0.0011	0.0011	0.0013
	Pyr (single)			
	Pyr	Pyr-Phe	Pyr-Ant	Pyr-Ant-Phe
	0.0036	0.0033	0.0044	0.0045

**Table 4:** Proton chemical shifts (ppm) for SDS without and with the solubilization of different PAHs at the SDS concentration of 120 mM.

Segment	Pure	Phe	Ant	Pyr	Phe-Ant	Phe-Pyr	Ant-Pyr	Phe-Ant-Pyr
S1	0.8972	0.8934	0.8972	0.8962	0.8929	0.8912	0.8954	0.8898
S2	1.3146	1.3042	1.3138	1.3089	1.3029	1.2934	1.3055	1.2917
S3	1.6889	1.6795	1.6883	1.6837	1.6783	1.6694	1.6805	1.6678
S4	4.0335	4.0283	4.0334	4.0308	4.0276	4.0236	4.0292	4.0219

## Data Availability

Data is contained within the article or supplementary material

## Supplementary Materials

The following are available online, Figure S1: ^1^H NMR spectra and peak assignment of the ternary Phe-Ant-Pyr system in 50 mM Tween 80 solution (A), the ternary Phe-Ant-Pyr system in CDCl_3_ solution (B) and the pure 50 mM Tween 80 solution (C); Figure S2: The line shape and chemical shift changes some Tw6 group protons (named as Tw6') compared with the pure Tween 80 after the solubilization of different PAHs solutes in 50 mM Tween 80 solutions; Figure S3: Relative chemical shifts (∆δ) of Phe (A), Ant (B) and Pyr (C) solubilized in different solubilization systems in 50 mM Tween 80 solution; Figure S4: Relative chemical shifts (∆δ) of Tween 80 protons as a function of concentration of Tween 80 in the single (Phe (A) and Ant (B)), binary (Phe-Ant (C), Phe-Pyr (D) and Ant-Pyr (E)) and ternary (Phe-Ant-Pyr (F)) solubilization systems at Tween 80 concentrations from 10 to 50 mM compared to those of the pure Tween 80 solution at the same concentration; Figure S5: Rela-tive chemical shifts (∆δ) of Tween 80 protons as a function of Savr in the single (Phe (A) and Ant (B)), binary (Phe-Ant (C), Phe-Pyr (D) and Ant-Pyr (E)) and ternary (Phe-Ant-Pyr (F)) solubiliza-tion systems at Tween 80 concentrations from 10 to 50 mM compared to those of the pure Tween 80 solution at the same concentration; Figure S6: ROESY spectrum of ternary Phe-Ant-Pyr system at the Tween 80 concentration of 50 mM with the mixing time of 0.2 s (its partially enlarged spec-trum is shown in Figure 6D); Figure S7: ^1^H NMR spectra and peak assignment of the ternary ter-nary Phe-Ant-Pyr system in 120 mM SDS solution (A), the ternary Phe-Ant-Pyr system in CDCl_3_ solution (B) and the pure 120 mM SDS solution (C); Figure S8: Relative chemical shifts (∆δ) of Phe (A), Ant (B) and Pyr (C) solubilized in different solubilization systems in 120 mM SDS solutions; Figure S9: Relative chemical shifts (∆δ) of SDS protons as a function of concentration of SDS in the single (Phe (A) and Ant (B)), binary (Phe-Ant (C), Phe-Pyr (D) and Ant-Pyr (E)) and ternary (Phe-Ant-Pyr (F)) solubilization systems at SDS concentrations from 40 to 120 mM compared to those of the pure SDS solution at the same concentration; Figure S10: Relative chemical shifts (∆δ) of SDS protons as a function of Savr in the single (Phe (A) and Ant (B)), binary (Phe-Ant (C), Phe-Pyr (D) and Ant-Pyr (E)) and ternary (Phe-Ant-Pyr (F)) solubilization systems at SDS concen-trations from 40 to 120 mM compared to those of the pure SDS solution at the same concentra-tion; Figure S11: NOESY spectrum of ternary Phe-Ant-Pyr system at the SDS concentration of 120 mM with the mixing time of 1 s (its partial enlarged spectrum is shown in Figure 12D).

## Sample Availability

Samples of the compounds are available from the authors.
