# Correction: Chen et al. The Total Solubility of the Co-Solubilized PAHs with Similar Structures Indicated by NMR Chemical Shift. *Molecules* 2021, *26*, 2793

**DOI:** 10.3390/molecules27061964

**Published:** 2022-03-18

**Authors:** Tao Chen, Xin Hu, Zhong Chen, Xiaohong Cui

**Affiliations:** Department of Electronic Science, Fujian Provincial Key Laboratory of Plasma and Magnetic Resonance, State Key Laboratory of Physical Chemistry of Solid Surfaces, Xiamen University, Xiamen 361005, China; 33320181150283@stu.xmu.edu.cn (T.C.); 33320191150287@stu.xmu.edu.cn (X.H.); chenz@xmu.edu.cn (Z.C.)

In the original article [1], there were several mistakes in the Abstract, References, and Supplementary Materials sections as published. In order to be easier for the reader to follow, a short piece of text from the original manuscript is cited together with the respective modification.

A part of the Abstract was missing and has been supplemented.

**In Abstract**

**Original:**

The synergism/inhibition level, solubilization sites and the total solubility (S_t_) of co-solubilization systems of phenanthrene, anthracene and pyrene in Tween 80 and sodium dodecyl sulfate (SDS) are studied by ^1^H-NMR, 2D nuclear overhauser effect spectroscopy (NOESY) and rotating frame overhauser effect spectroscopy (ROESY). In Tween 80, inhibition for phenanthrene, anthracene and pyrene is observed in most binary and ternary systems. However, in SDS, synergism is predominant. After analysis, we find that the different synergism or inhibition situation between Tween 80 and SDS is related to the different types of surfactants used and the resulting different co-solubilization mechanisms. In addition, we also find that three polycyclic aromatic hydrocarbons.

**This should be replaced with the following:**

The synergism/inhibition level, solubilization sites and the total solubility (S_t_) of co-solubilization systems of phenanthrene, anthracene and pyrene in Tween 80 and sodium dodecyl sulfate (SDS) are studied by ^1^H-NMR, 2D nuclear overhauser effect spectroscopy (NOESY) and rotating frame overhauser effect spectroscopy (ROESY). In Tween 80, inhibition for phenanthrene, anthracene and pyrene is observed in most binary and ternary systems. However, in SDS, synergism is predominant. After analysis, we find that the different synergism or inhibition situation between Tween 80 and SDS is related to the different types of surfactants used and the resulting different co-solubilization mechanisms. In addition, we also find that three polycyclic aromatic hydrocarbons (PAHs) have similar solubilization sites in both Tween 80 and SDS, which are almost unchanged in co-solubilization systems. Due to the similar solubilization sites, the chemical shift changes of surfactant and PAH protons follow the same pattern in all solubilization systems, and the order of chemical shift changes is consistent with the order of changes in the S_t_ of PAHs. In this case, it is feasible to evaluate S_t_ of PAHs by chemical shift. In both Tween 80 and SDS solutions, the ternary solubilization system has relatively high S_t_ rankings. Therefore, in practical applications, a good overall solubilization effect can be expected. 

**Incorrect Reference**

Reference “Bernardez, L.A. Investigation on the locus of solubilization of polycyclic aromatic hydrocarbons in non-ionic surfactant micelles with 1H NMR spectroscopy. *Colloid Surf. A-Physicochem. Eng. Asp.* **2008**, *324*, 71–78, https://doi.org/10.1016/j.colsurfa.2008.03.027.” was missing in the original article. Therefore, the involved citations and references have been revised accordingly.

**In Section 2.1.1. Apparent Solubility**

**Original:**

Scheme 1 shows the chemical structures and proton numbering of surfactants and three PAHs of Phe, Ant and Pyr. The represented ^1^H-NMR spectra of ternary PAHs solubilized in 50 mM Tween 80 micellar solutions and pure ternary PAHs solubilized in chloroform and pure 50 mM Tween 80 in D_2_O as references are shown in Figure S1, Supplementary Materials. Based on the integral areas of resonances Ph5, An3 and Py2 relative to that of TSP with the known concentration of 2.82 mM, the actual apparent concentrations of three PAHs were obtained. The apparent solubility of each PAHs in single, binary and ternary solubilization systems as a function of the Tween 80 concentration are shown in Figure 1. It can be found that all the solubilities of Phe, Ant and Pyr increase linearly with the Tween 80 concentration, whenever they are solubilized in single or combined systems. Molar solubilization ratio (*MSR*) is used to evaluate the solubilization potential of a surfactant, which is the molar concentration of the solubilized compound per mole of the surfactant concentration when the surfactant concentration is above CMC [36,37], expressed by

(1) $$MSR=\frac{S-S_{CMC}}{C-\mathrm{CMC}}$$

where *S* is the apparent solubility of a solute at the surfactant concentration of *C*, *S_CMC_* is the apparent solubility of the solute at CMC. Usually, the slope of the solubility with respect to the surfactant concentration can be considered to be *MSR*. According to the linear fitting curves, *MSR* values of three PAHs in single, binary and ternary solubilization systems in Tween 80 solutions can be obtained and shown in Table 1. *MSR* values of single Phe, Ant and Pyr are 0.1170, 0.0094 and 0.0468, respectively. Accordingly, single Phe has the strongest solubilization ability, followed by single Pyr, and finally Ant, which are in accordance with the order of their aqueous solubility of 5.55 μM [38], 0.684 μM [39] and 0.268 μM [24]. The aqueous solubility is closely related to their structures. Phe and Ant are isomers; however, because three benzene rings of Ant are in a line, therefore Ant exhibits weaker polarity than Phe. For Pyr, there is one more benzene ring than Phe. Hence, its polarity is also weaker than that of Phe. The above single solubility results indicate that the solubility of PAHs in micellar solutions mainly depends on its aqueous solubility.

**This should be replaced with the following:**

Scheme 1 shows the chemical structures and proton numbering of surfactants and three PAHs of Phe, Ant and Pyr. The represented ^1^H-NMR spectra of ternary PAHs solubilized in 50 mM Tween 80 micellar solutions and pure ternary PAHs solubilized in chloroform and pure 50 mM Tween 80 in D_2_O as references are shown in Figure S1, Supplementary Materials. Based on the integral areas of resonances Ph5, An3 and Py2 relative to that of TSP with the known concentration of 2.82 mM, the actual apparent concentrations of three PAHs were obtained. The apparent solubility of each PAHs in single, binary and ternary solubilization systems as a function of the Tween 80 concentration are shown in Figure 1, Supplementary Materials. It can be found that all the solubilities of Phe, Ant and Pyr increase linearly with the Tween 80 concentration, whenever they are solubilized in single or combined systems. Molar solubilization ratio (*MSR*) is used to evaluate the solubilization potential of a surfactant, which is the molar concentration of the solubilized compound per mole of the surfactant concentration when the surfactant concentration is above CMC [37,38], expressed by:

(1) $$MSR=\frac{S-S_{CMC}}{C-\mathrm{CMC}}$$

where *S* is the apparent solubility of a solute at the surfactant concentration of *C*, S*_CMC_* is the apparent solubility of the solute at CMC. Usually, the slope of the solubility with respect to the surfactant concentration can be considered to be *MSR*. According to the linear fitting curves, *MSR* values of three PAHs in single, binary and ternary solubilization systems in Tween 80 solutions can be obtained and shown in Table 1. *MSR* values of single Phe, Ant and Pyr are 0.1170, 0.0094 and 0.0468, respectively. Accordingly, single Phe has the strongest solubilization ability, followed by single Pyr, and finally Ant, which are in accordance with the order of their aqueous solubility of 5.55 μM [39], 0.684 μM [40] and 0.268 μM [24]. The aqueous solubility is closely related to their structures. Phe and Ant are isomers; however, because three benzene rings of Ant are in a line, therefore Ant exhibits weaker polarity than Phe. For Pyr, there is one more benzene ring than Phe. Hence, its polarity is also weaker than that of Phe. The above single solubility results indicate that the solubility of PAHs in micellar solutions mainly depends on its aqueous solubility.

**Original:**

Saumyen Guha et al. [40] found that: if PAHs compete with each other for the internal position of micelles, the solubility of one or more PAHs in the presence of other PAHs will be reduced. 

**This should be replaced with the following:**

Saumyen Guha et al. [41] found that: if PAHs compete with each other for the internal position of micelles, the solubility of one or more PAHs in the presence of other PAHs will be reduced.

**In Section 3.1. Materials**

**Original:**

First, a series of Tween 80 pure solutions at concentrations of 10, 20, 30, 40 and 50 mM and SDS pure solutions at concentrations of 40, 60, 80, 100 and 120 mM were prepared in D_2_O, respectively. These concentrations were set higher than their CMCs [32,41]. 

**This should be replaced with the following:**

First, a series of Tween 80 pure solutions at concentrations of 10, 20, 30, 40 and 50 mM and SDS pure solutions at concentrations of 40, 60, 80, 100 and 120 mM were prepared in D_2_O, respectively. These concentrations were set higher than their CMCs [32,42].

A part of the content in supporting materials was missing and has been supplemented.

**Original:**

**Supplementary Materials:** The following are available online: Figures S1–S11. It includes the 1H-NMR spectra of two surfactants and three PAHs, the chemical shift changes of PAHs in different systems, relative chemical shift of Tween 80 and SDS protons in different PAHs systems, and the full spectrum of NOESY in Tween 80 and SDS of the ternary PAHs system.

**This should be replaced with the following:**

**Supplementary Materials:** The following are available online: Figure S1: ^1^H NMR spectra and peak assignment of the ternary Phe-Ant-Pyr system in 50 mM Tween 80 solution (A), the ternary Phe-Ant-Pyr system in CDCl_3_ solution (B) and the pure 50 mM Tween 80 solution (C); Figure S2: The line shape and chemical shift changes some Tw6 group protons (named as Tw6’) compared with the pure Tween 80 after the solubilization of different PAHs solutes in 50 mM Tween 80 solutions; Figure S3: Relative chemical shifts (∆δ) of Phe (A), Ant (B) and Pyr (C) solubilized in different solubilization systems in 50 mM Tween 80 solution; Figure S4: Relative chemical shifts (∆δ) of Tween 80 protons as a function of concentration of Tween 80 in the single (Phe (A) and Ant (B)), binary (Phe-Ant (C), Phe-Pyr (D) and Ant-Pyr (E)) and ternary (Phe-Ant-Pyr (F)) solubilization systems at Tween 80 concentrations from 10 to 50 mM compared to those of the pure Tween 80 solution at the same concentration; Figure S5: Relative chemical shifts (∆δ) of Tween 80 protons as a function of S_avr_ in the single (Phe (A) and Ant (B)), binary (Phe-Ant (C), Phe-Pyr (D) and Ant-Pyr (E)) and ternary (Phe-Ant-Pyr (F)) solubilization systems at Tween 80 concentrations from 10 to 50 mM compared to those of the pure Tween 80 solution at the same concentration; Figure S6: ROESY spectrum of ternary Phe-Ant-Pyr system at the Tween 80 concentration of 50 mM with the mixing time of 0.2 s (its partially enlarged spectrum is shown in Figure 6D); Figure S7: ^1^H NMR spectra and peak assignment of the ternary ternary Phe-Ant-Pyr system in 120 mM SDS solution (A), the ternary Phe-Ant-Pyr system in CDCl_3_ solution (B) and the pure 120 mM SDS solution (C); Figure S8: Relative chemical shifts (∆δ) of Phe (A), Ant (B) and Pyr (C) solubilized in different solubilization systems in 120 mM SDS solutions; Figure S9: Relative chemical shifts (∆δ) of SDS protons as a function of concentration of SDS in the single (Phe (A) and Ant (B)), binary (Phe-Ant (C), Phe-Pyr (D) and Ant-Pyr (E)) and ternary (Phe-Ant-Pyr (F)) solubilization systems at SDS concentrations from 40 to 120 mM compared to those of the pure SDS solution at the same concentration; Figure S10: Relative chemical shifts (∆δ) of SDS protons as a function of S_avr_ in the single (Phe (A) and Ant (B)), binary (Phe-Ant (C), Phe-Pyr (D) and Ant-Pyr (E)) and ternary (Phe-Ant-Pyr (F)) solubilization systems at SDS concentrations from 40 to 120 mM compared to those of the pure SDS solution at the same concentration; Figure S11: NOESY spectrum of ternary Phe-Ant-Pyr system at the SDS concentration of 120 mM with the mixing time of 1 s (its partial enlarged spectrum is shown in Figure 12D).

**In References**

**Original:**

35. Ashraf, U.; Lone, M.S.; Masrat, R.; Shah, R.A.; Afzal, S.; Chat, O.A.; Dar, A.A. Co-solubilization of polycyclic aromatic hydrocarbon mixtures in aqueous micellar systems and its correlation with FRET for enhanced remediation processes. *Chemosphere* **2020**, *242*, 125160, https://doi.org/10.1016/j.chemosphere.2019.125160.
36. Edwards, D.A.; Luthy, R.G.; Liu, Z. Solubilization of polycyclic aromatic hydrocarbons in micellar nonionic surfactant solutions. *Environ. Sci. Technol.* **1991**, *25*, 127–133, https://doi.org/10.1021/es00013a014.
37. Peng, S.; Wu, W.; Chen, J. Removal of PAHs with surfactant-enhanced soil washing: Influencing factors and removal effectiveness. *Chemosphere* **2011**, *82*, 1173–1177, https://doi.org/10.1016/j.chemosphere.2010.11.076.
38. Kuramochi, H.; Nakajima, D.; Goto, S.; Kawamoto, K.; Maeda, K. Water solubility of solid solution of phenanthrene and anthracene mixture. *Polycycl. Aromat. Compd.* **2006**, *26*, 299–312, https://doi.org/10.1080/10406630600930005.
39. Pearlman, R.S.; Yalkowsky, S.H.; Banerjee, S. Water solubilities of polynuclear aromatic and heteroaromatic compounds. *J. Phys. Chem. Ref. Data* **1984**, *13*, 555–562, https://doi.org/10.1063/1.555712.
40. Guha, S.; Jaffe, P.R.; Peters, C.A. Solubilization of PAH mixtures by a nonionic surfactant. *Environ. Sci. Technol.* **1998**, *32*, 930–935, https://doi.org/10.1021/es970695c.
41. Haque, M.E.; Das, A.R.; Moulik, S.P. Mixed micelles of sodium deoxycholate and polyoxyethylene sorbitan monooleate (Tween 80). *J. Colloid Interface Sci.* **1999**, *217*, 1–7, https://doi.org/10.1006/jcis.1999.6267.

This should be replaced with:

35. Ashraf, U.; Lone, M.S.; Masrat, R.; Shah, R.A.; Afzal, S.; Chat, O.A.; Dar, A.A. Co-solubilization of polycyclic aromatic hydrocarbon mixtures in aqueous micellar systems and its correlation with FRET for enhanced remediation processes. *Chemosphere* **2020**, *242*, 125160, https://doi.org/10.1016/j.chemosphere.2019.125160.
36. Bernardez, L.A. Investigation on the locus of solubilization of polycyclic aromatic hydrocarbons in non-ionic surfactant micelles with ^1^H NMR spectroscopy. *Colloid Surf. A-Physicochem. Eng. Asp.* **2008**, *324*, 71–78, https://doi.org/10.1016/j.colsurfa.2008.03.027.
37. Edwards, D.A.; Luthy, R.G.; Liu, Z. Solubilization of polycyclic aromatic hydrocarbons in micellar nonionic surfactant solutions. *Environ. Sci. Technol.* **1991**, *25*, 127–133, https://doi.org/10.1021/es00013a014.
38. Peng, S.; Wu, W.; Chen, J. Removal of PAHs with surfactant-enhanced soil washing: Influencing factors and removal effectiveness. *Chemosphere* **2011**, *82*, 1173–1177, https://doi.org/10.1016/j.chemosphere.2010.11.076.
39. Kuramochi, H.; Nakajima, D.; Goto, S.; Kawamoto, K.; Maeda, K. Water solubility of solid solution of phenanthrene and anthracene mixture. *Polycycl. Aromat. Compd.* **2006**, *26*, 299–312, https://doi.org/10.1080/10406630600930005.
40. Pearlman, R.S.; Yalkowsky, S.H.; Banerjee, S. Water solubilities of polynuclear aromatic and heteroaromatic compounds. *J. Phys. Chem. Ref. Data* **1984**, *13*, 555–562, https://doi.org/10.1063/1.555712.
41. Guha, S.; Jaffe, P.R.; Peters, C.A. Solubilization of PAH mixtures by a nonionic surfactant. *Environ. Sci. Technol.* **1998**, *32*, 930–935, https://doi.org/10.1021/es970695c.
42. Haque, M.E.; Das, A.R.; Moulik, S.P. Mixed micelles of sodium deoxycholate and polyoxyethylene sorbitan monooleate (Tween 80). *J. Colloid Interface Sci.* **1999**, *217*, 1–7, https://doi.org/10.1006/jcis.1999.6267.

The original article has been updated. These changes have no material impact on the aim of our paper. We apologize for any inconvenience to the readers.
