# Supplementary material for: The Total Solubility of the Co-Solubilized PAHs with Similar Structures Indicated by NMR Chemical Shift
Source: Molecules. 2021 May 10;26(9):2793. doi: 10.3390/molecules26092793 (PMC8125976; doi:10.3390/molecules26092793)
Supplement: Supplementary file 1 [file molecules-26-02793-s001.zip › molecules-1178761-supplementary.pdf]

Supplementary Material

The Total Solubility of the Co-Solubilized PAHs with Similar Structures Indicated by NMR Chemical Shift

Tao Chen, Xin Hu, Zhong Chen and Xiaohong Cui *

Department of Electronic Science, Fujian Provincial Key Laboratory of Plasma and Magnetic Resonance, State Key Laboratory of Physical Chemistry of Solid Surfaces, Xiamen University, Xiamen, China; 33320181150283@stu.xmu.edu.cn (T.C.); 33320191150287@stu.xmu.edu.cn (X.H.); chenz@xmu.edu.cn (Z.C.)

* Correspondence: cuixh@xmu.edu.cn Tel.: +86-189-0022-9869

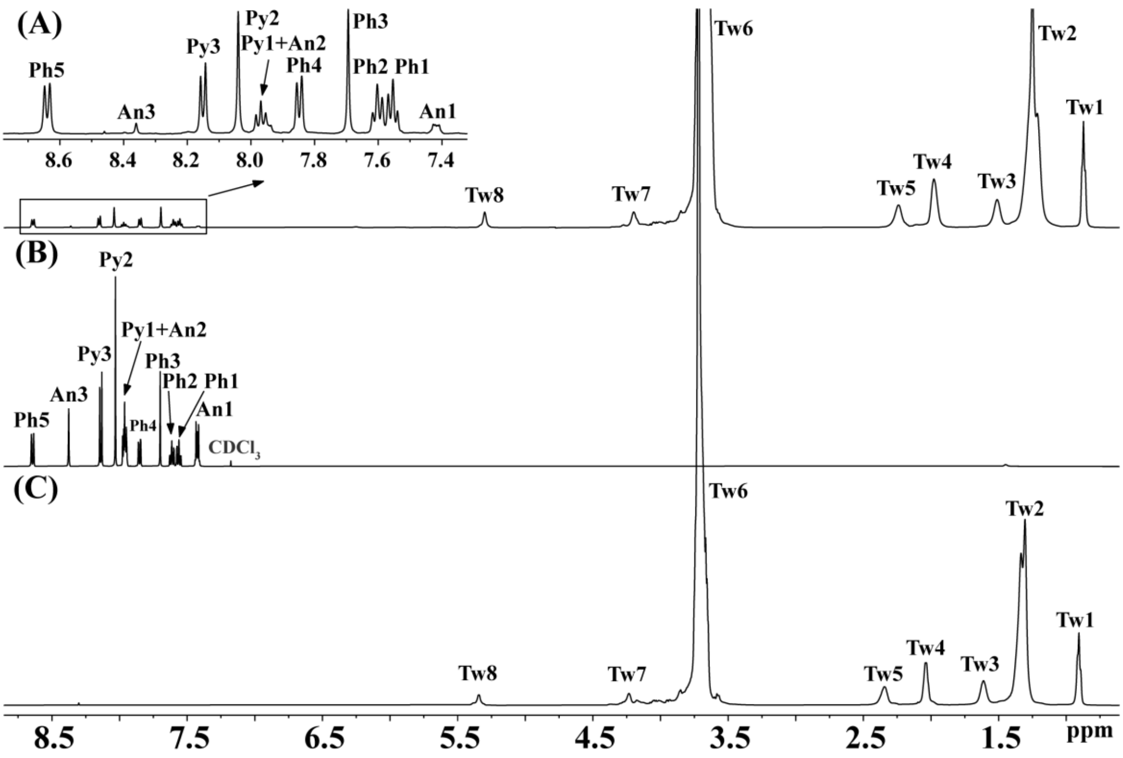

Figure S1. ^1H NMR spectra and peak assignment of the ternary Phe-Ant-Pyr system in 50 mM Tween 80 solution (A), the ternary Phe-Ant-Pyr system in $CDCl_3$ solution (B) and the pure 50 mM Tween 80 solution (C).

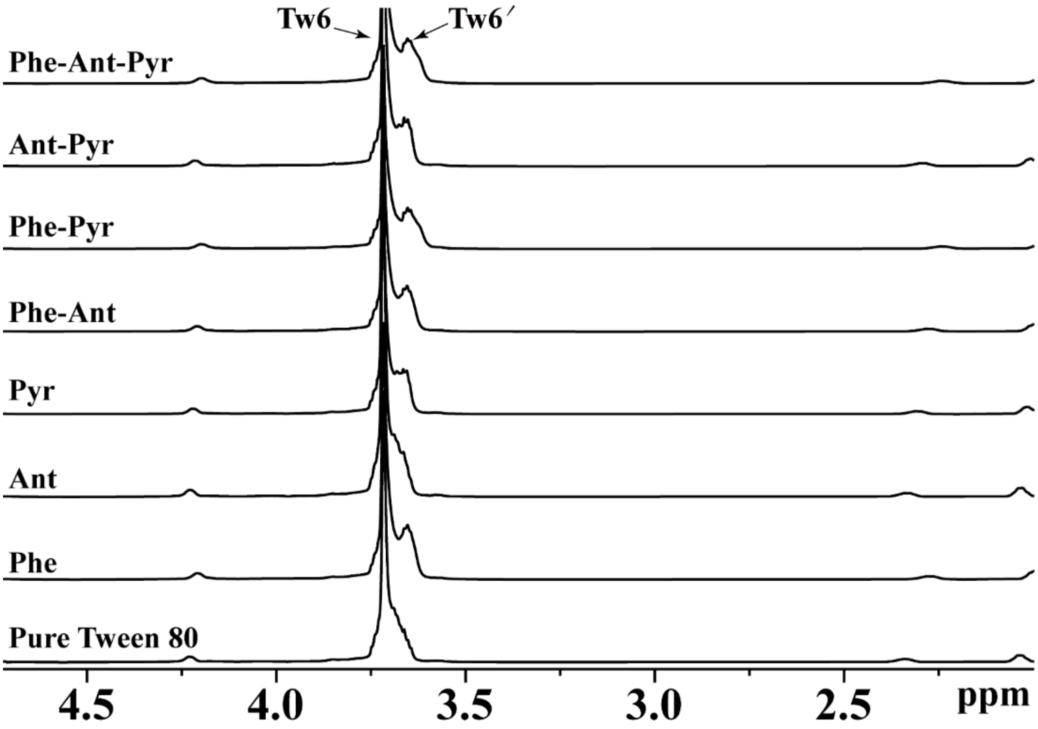

Figure S2. The line shape and chemical shift changes some Tw6 group protons (named as Tw6') compared with the pure Tween 80 after the solubilization of different PAHs solutes in 50 mM Tween 80 solutions.

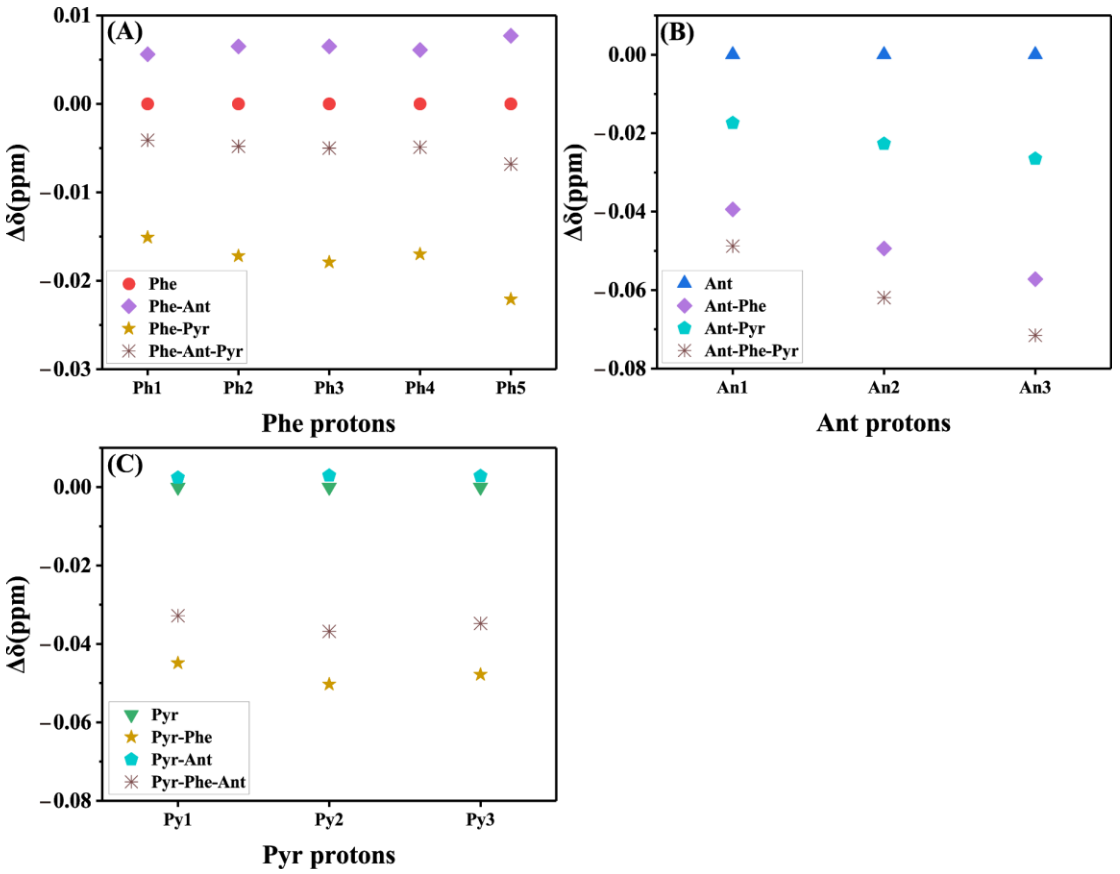

Figure S3. Relative chemical shifts (Δδ) of Phe (A), Ant (B) and Pyr (C) solubilized in different solubilization systems in 50 mM Tween 80 solution.

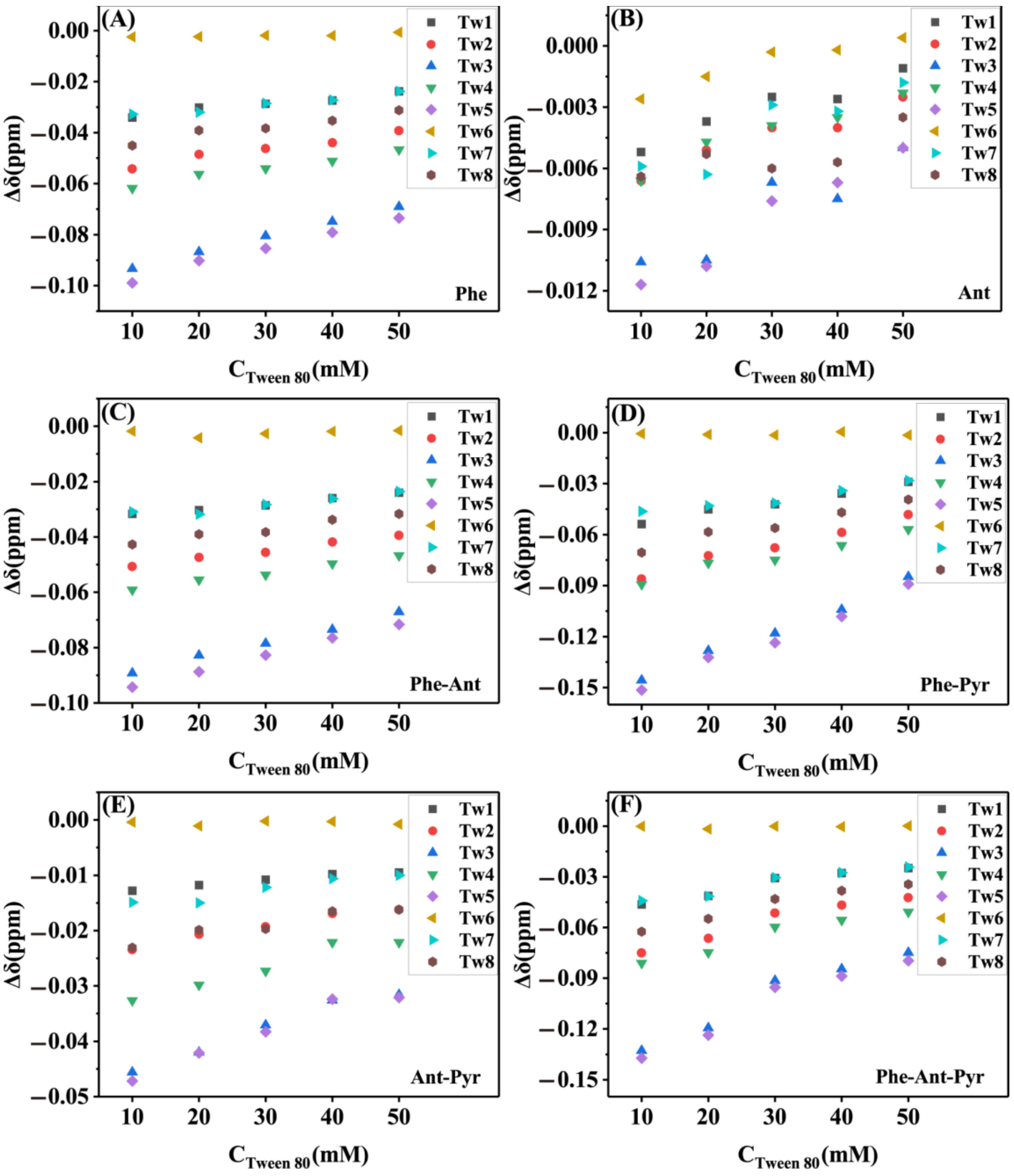

Figure S4. Relative chemical shifts (Δδ) of Tween 80 protons as a function of concentration of Tween 80 in the single (Phe (A) and Ant (B)), binary (Phe-Ant (C), Phe-Pyr (D) and Ant-Pyr (E)) and ternary (Phe-Ant-Pyr (F)) solubilization systems at Tween 80 concentrations from 10 to 50 mM compared to those of the pure Tween 80 solution at the same concentration.

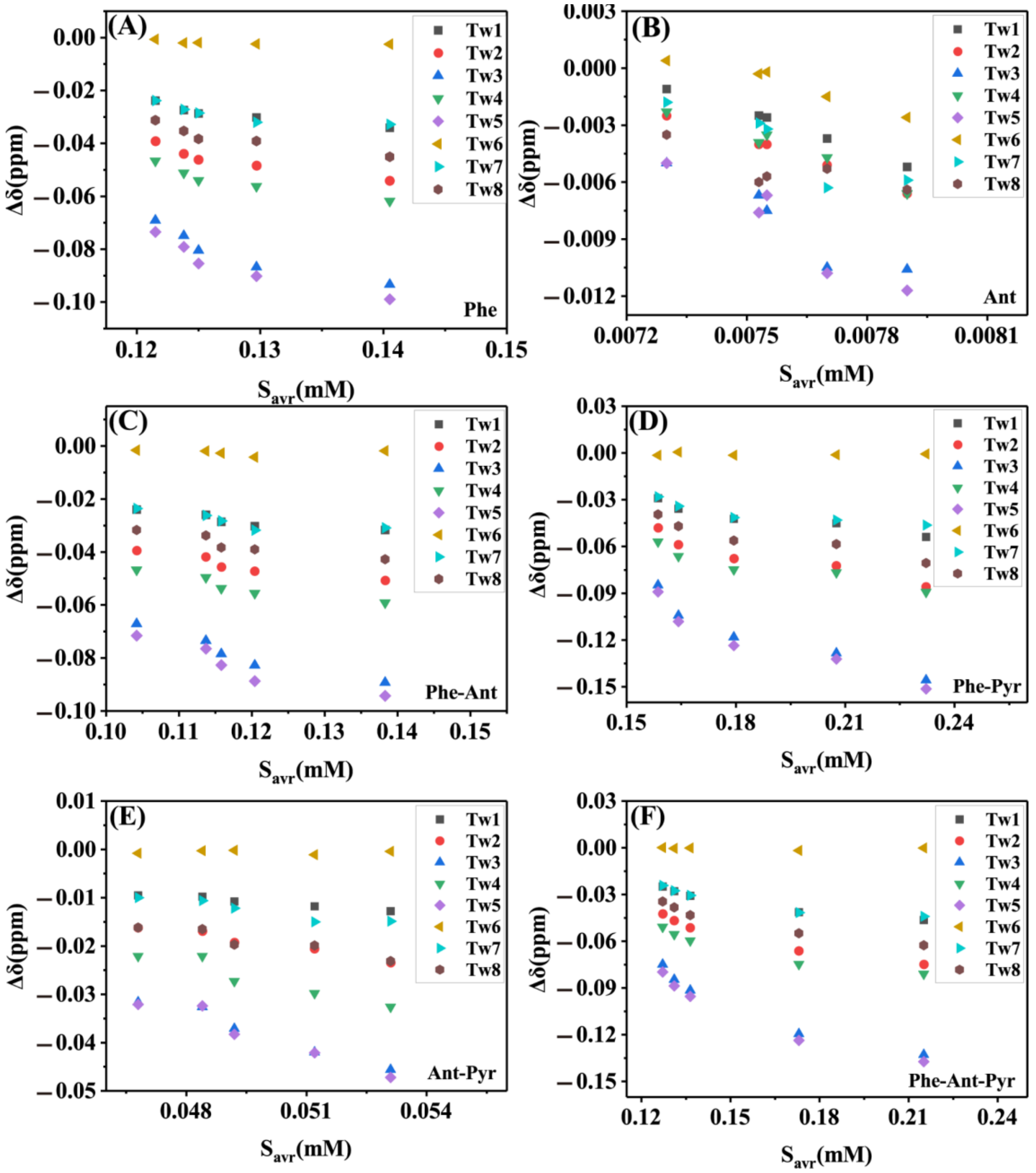

Figure S5. Relative chemical shifts (Δδ) of Tween 80 protons as a function of S_{avr} in the single (Phe (A) and Ant (B)), binary (Phe-Ant (C), Phe-Pyr (D) and Ant-Pyr (E)) and ternary (Phe-Ant-Pyr (F)) solubilization systems at Tween 80 concentrations from 10 to 50 mM compared to those of the pure Tween 80 solution at the same concentration.

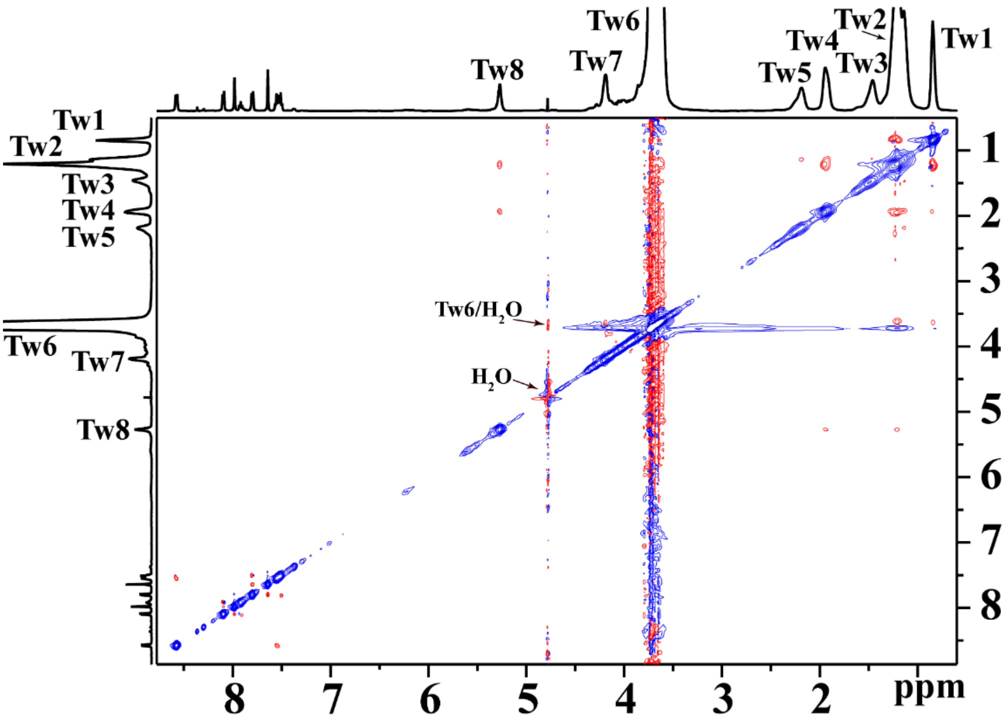

Figure S6. ROESY spectrum of ternary Phe-Ant-Pyr system at the Tween 80 concentration of 50 mM with the mixing time of 0.2 s. Its partially enlarged spectrum is shown in Figure 6D.

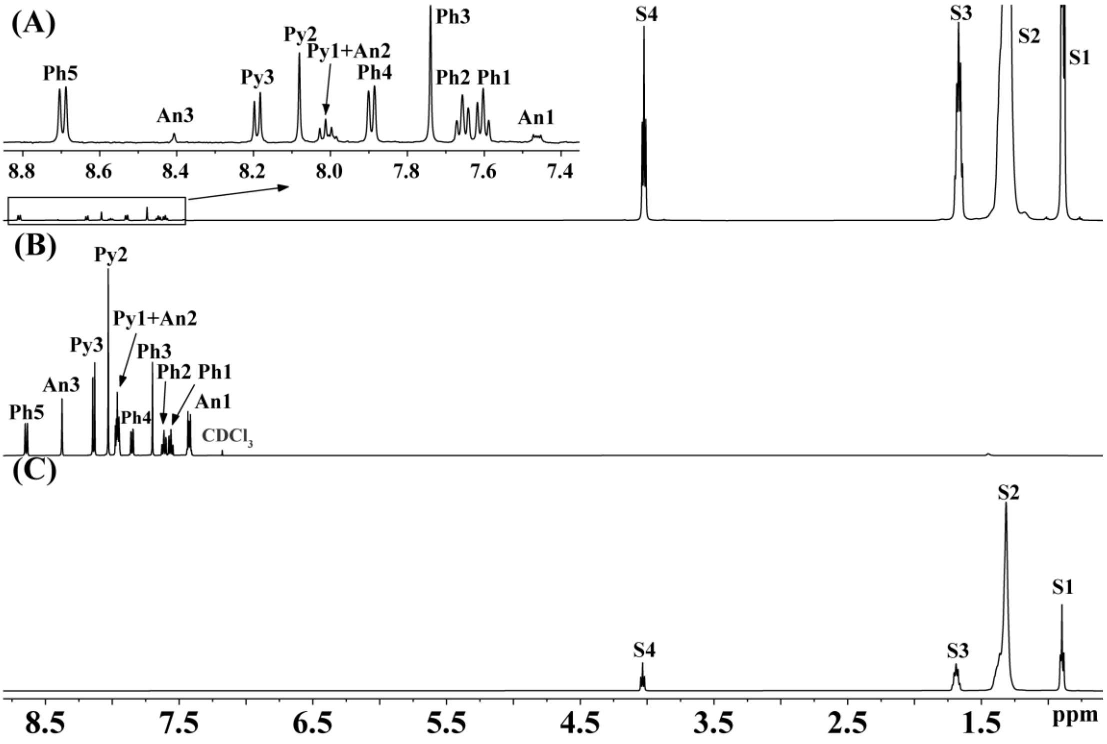

Figure S7. ^{1}H NMR spectra and peak assignment of the ternary ternary Phe-Ant-Pyr system in 120 mM SDS solution (A), the ternary Phe-Ant-Pyr system in $CDCl_3$ solution (B) and the pure 120 mM SDS solution (C).

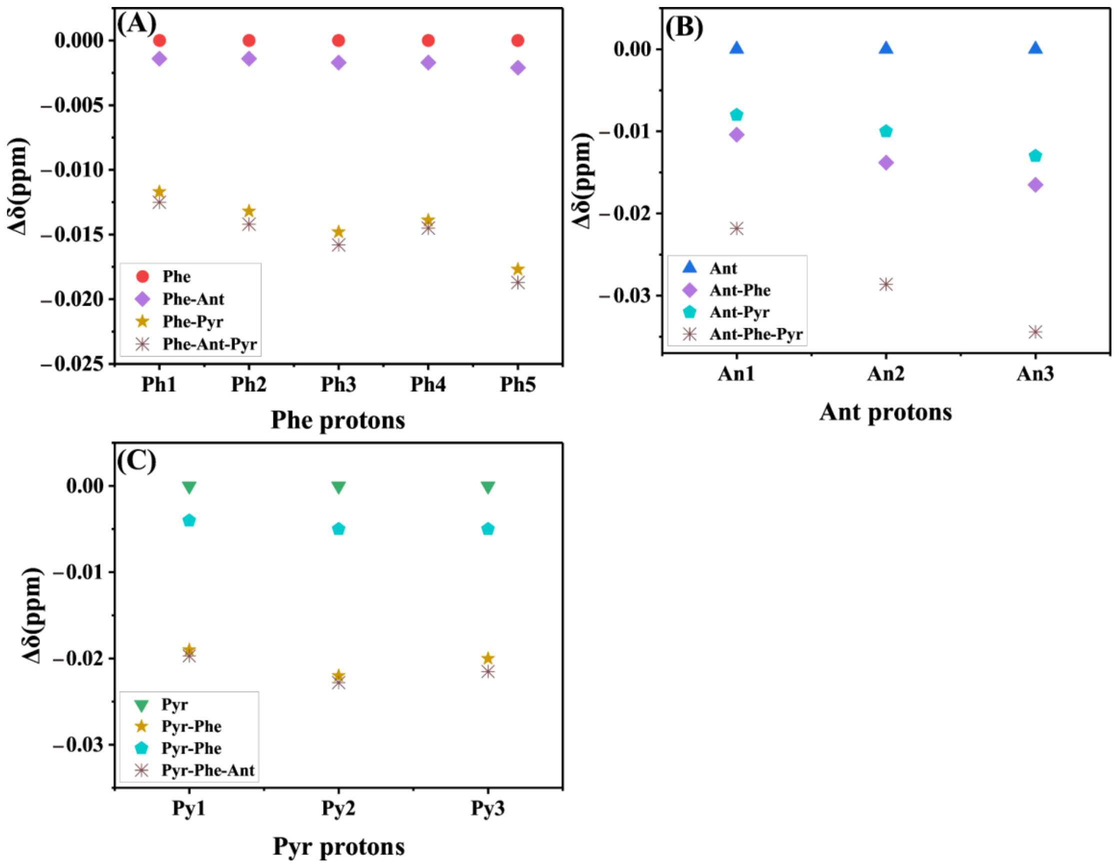

Figure S8. Relative chemical shifts (Δδ) of Phe (A), Ant (B) and Pyr (C) solubilized in different solubilization systems in 120 mM SDS solutions.

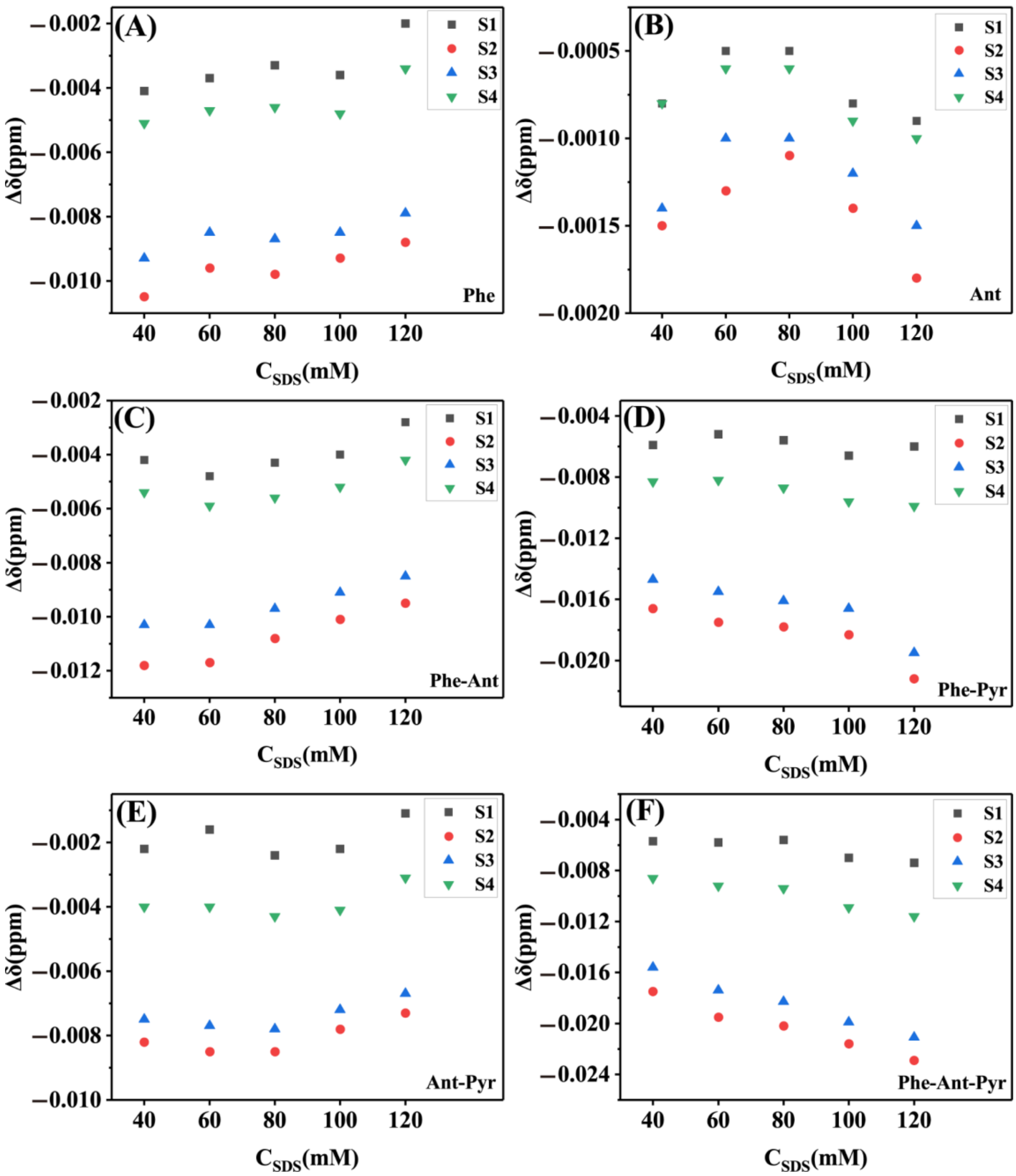

Figure S9. Relative chemical shifts (Δδ) of SDS protons as a function of concentration of SDS in the single (Phe (A) and Ant (B)), binary (Phe-Ant (C), Phe-Pyr (D) and Ant-Pyr (E)) and ternary (Phe-Ant-Pyr (F)) solubilization systems at SDS concentrations from 40 to 120 mM compared to those of the pure SDS solution at the same concentration.

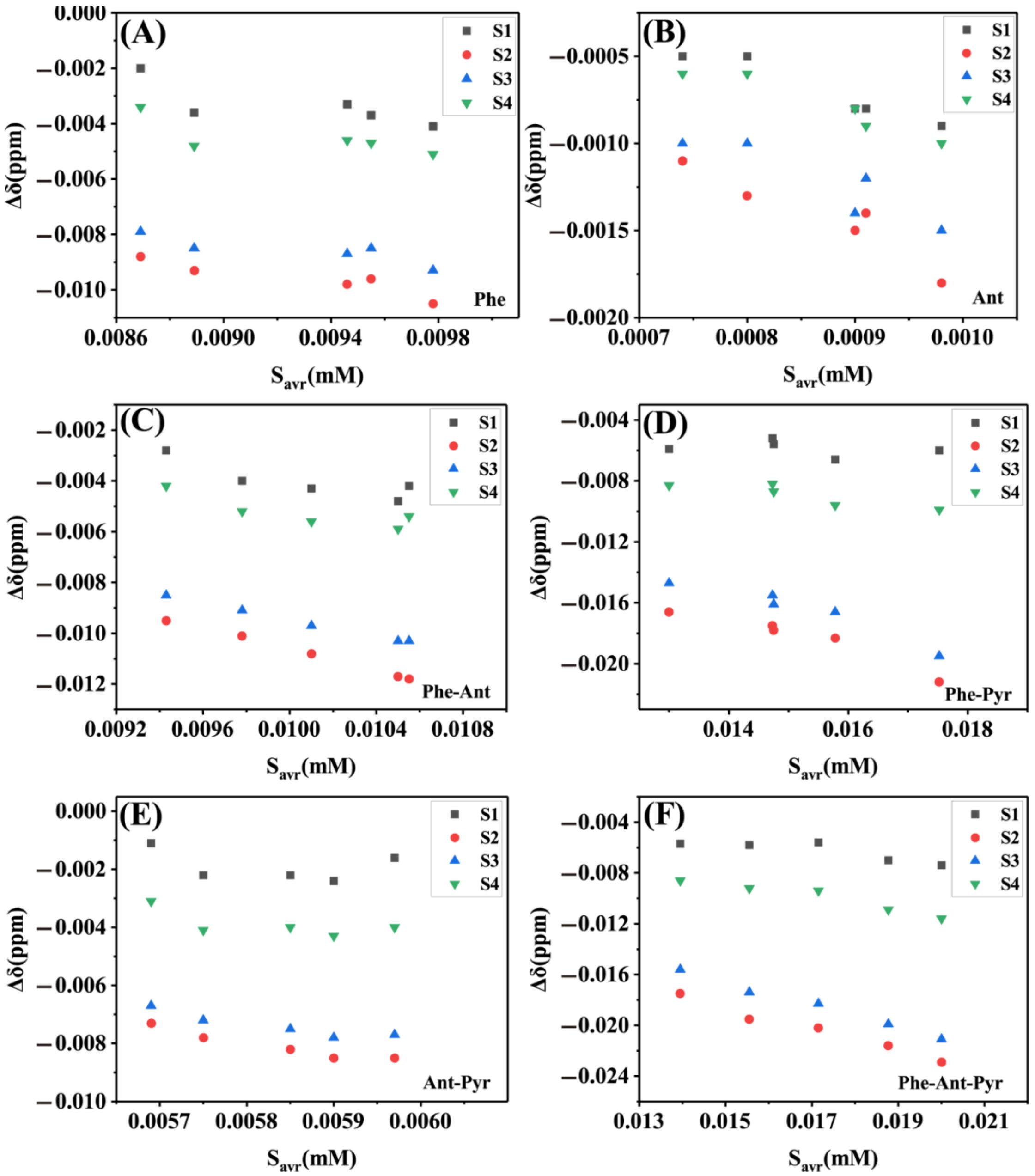

Figure S10. Relative chemical shifts (Δδ) of SDS protons as a function of S_{avr} in the single (Phe (A) and Ant (B)), binary (Phe-Ant (C), Phe-Pyr (D) and Ant-Pyr (E)) and ternary (Phe-Ant-Pyr (F)) solubilization systems at SDS concentrations from 40 to 120 mM compared to those of the pure SDS solution at the same concentration.

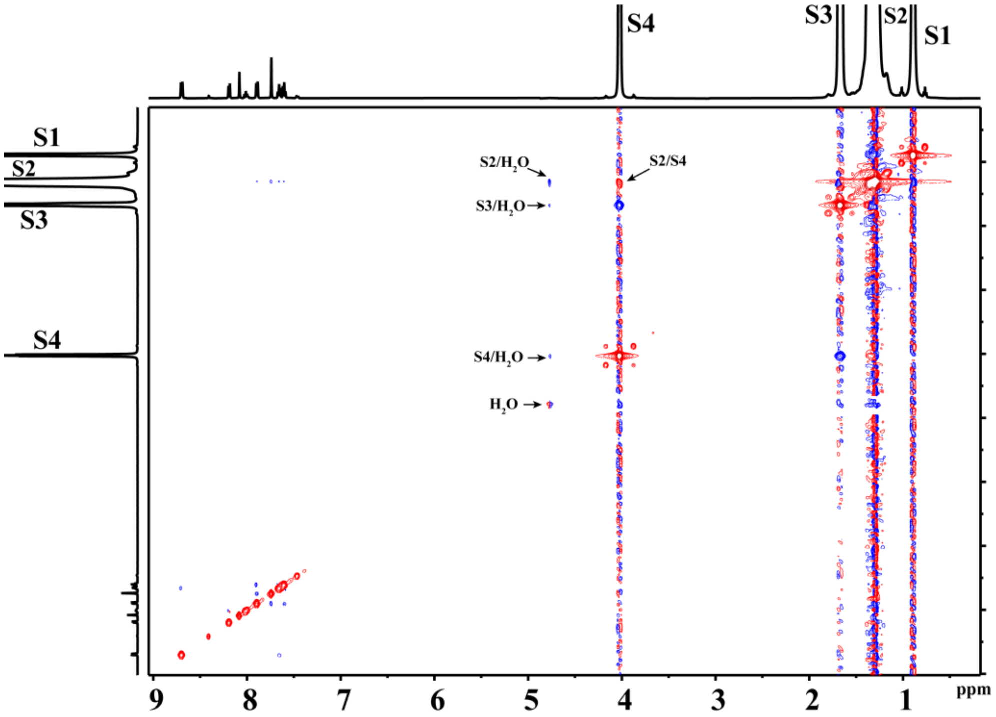

Figure S11. NOESY spectrum of ternary Phe-Ant-Pyr system at the SDS concentration of 120 mM with the mixing time of 1 s. Its partial enlarged spectrum is shown in Figure 12D.
